# A Solvent-Free Covalent Organic Framework Single-Ion Conductor Based on Ion–Dipole Interaction for All-Solid-State Lithium Organic Batteries

**DOI:** 10.1007/s40820-024-01485-3

**Published:** 2024-08-09

**Authors:** Zhongping Li, Kyeong-Seok Oh, Jeong-Min Seo, Wenliang Qin, Soohyoung Lee, Lipeng Zhai, Changqing Li, Jong-Beom Baek, Sang-Young Lee

**Affiliations:** 1 Department of Chemical and Biomolecular Engineering, Yonsei University, 50 Yonsei-Ro, Seodaemun-Gu, Seoul, 03722 Republic of Korea; 2 School of Energy and Chemical Engineering, Ulsan National Institute of Science and Technology (UNIST), 50 UNIST-Gil, Eonyang-Eup, Ulju-Gun, Ulsan, 44919 Republic of Korea; 3 Henan Key Laboratory of Functional Salt Materials, Center for Advanced Materials Research, Zhongyuan University of Technology, Zhengzhou, 450007 People’s Republic of China; 4 Department of Battery Conflation Engineering, Yonsei University, 50, Yonsei-Ro, Seodaemun-Gu, Seoul, 03772 Republic of Korea; 5 Department of Battery Engineering, Yonsei University, 50, Yonsei-Ro, Seodaemun-Gu, Seoul, 03772 Republic of Korea

**Keywords:** Solid organic single-ion conductors, Solvent-free covalent organic frameworks, All-solid-state Li organic batteries, Ion–dipole interaction, Pore functionalization

## Abstract

Single-ion conductors based on covalent organic frameworks (COFs) have garnered attention as a potential alternative to currently prevalent inorganic ion conductors owing to their structural uniqueness and chemical versatility. However, the sluggish Li^+^ conduction has hindered their practical applications. Here, we present a class of solvent-free COF single-ion conductors (Li-COF@P) based on weak ion–dipole interaction as opposed to traditional strong ion–ion interaction. The ion (Li^+^ from the COF)–dipole (oxygen from poly(ethylene glycol) diacrylate embedded in the COF pores) interaction in the Li-COF@P promotes ion dissociation and Li^+^ migration via directional ionic channels. Driven by this single-ion transport behavior, the Li-COF@P enables reversible Li plating/stripping on Li-metal electrodes and stable cycling performance (88.3% after 2000 cycles) in organic batteries (Li metal anode||5,5’-dimethyl-2,2’-bis-p-benzoquinone (Me_2_BBQ) cathode) under ambient operating conditions, highlighting the electrochemical viability of the Li-COF@P for all-solid-state organic batteries.

**Supplementary Information:**

The online version contains supplementary material available at 10.1007/s40820-024-01485-3.

## Highlights

1. A class of solvent-free covalent organic framework (COF) single-ion conductors (Li-COF@P) has been designed via ion–dipole interaction as opposed to traditional ion–ion interaction, promoting ion dissociation and Li^+^ migration through directional ionic channels.
2. The Li-COF@P enabled long cycle life (88.3% after 2000 cycles) in all-solid-state Li organic batteries (ASSLOBs) under ambient operating conditions, which outperformed those of previously reported ASSOLBs.
3. This Li-COF@P strategy holds promise as a viable alternative to the currently prevalent inorganic solid electrolytes.

**Supplementary Information:**

The online version contains supplementary material available at 10.1007/s40820-024-01485-3.

## Graphical abstract

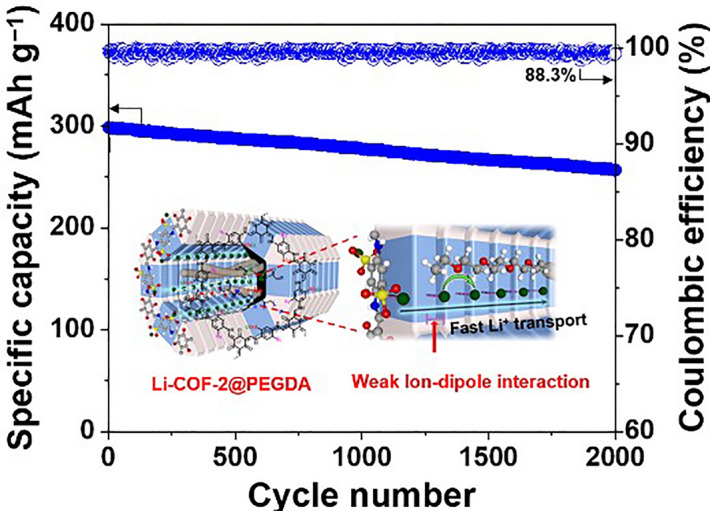

## Introduction

Ion conductors play a pivotal role in determining the redox reaction kinetics of electrochemical energy storage systems [1–3]. This significance has catalyzed the exploration of advanced ion conductors that afford high ionic conductivity and electrochemical stability with electrode materials. Despite the widespread use of commercial liquid electrolytes in lithium (Li)-ion batteries (LIBs) [4, 5], the presence of freely mobile anions and organic solvents in the electrolytes tends to cause inhomogeneous ion flux and undesirable side reactions with electrode materials, resulting in the performance degradation and safety failure of the batteries [6, 7]. Enormous efforts have been devoted to address these issues with a focus on single Li^+^ conductors for all-solid state Li batteries [8, 9], which have been investigated as a promising candidate for post LIBs owing to their high energy density and safety gain.

Previous studies on solid single Li^+^ conductors have focused on the design of immobilized anionic domains, such as inorganic lattices (including oxides and sulfides) and polyanions [10, 11]. However, their intrinsically anionic moieties tightly bind to Li^+^ via strong ion–ion attraction. Moreover, these electrolytes often provide random and reticulated pathways for ion conduction [10–12]. Recently, a new concept of solid single Li^+^ conductors based on covalent organic frameworks (COFs) [13–16] was reported as an attractive alternative owing to their one-dimensional (1D) directional ion conduction channels and versatile chemical structure [17–29]. Anionic frameworks were introduced into most of the previously reported COF ion conductors to provide high cationic transference number (*t*_Li_^+^) [30–33]; however, they have suffered from insufficient ionic conductivities and high activation energies for ion conduction because of the strong Li^+^ binding energies caused by the ion (Li^+^)–ion (anionic framework of COF) interaction. In addition to this ion transport issue, solid single Li^+^ conductors should fulfill the mechanical requirements to ensure their role as ion-conducting membranes. However, most COFs are microcrystal-based powders, which hinder their fabrication into practical thin and flexible films [17, 34–36].

**Fig. 1:**
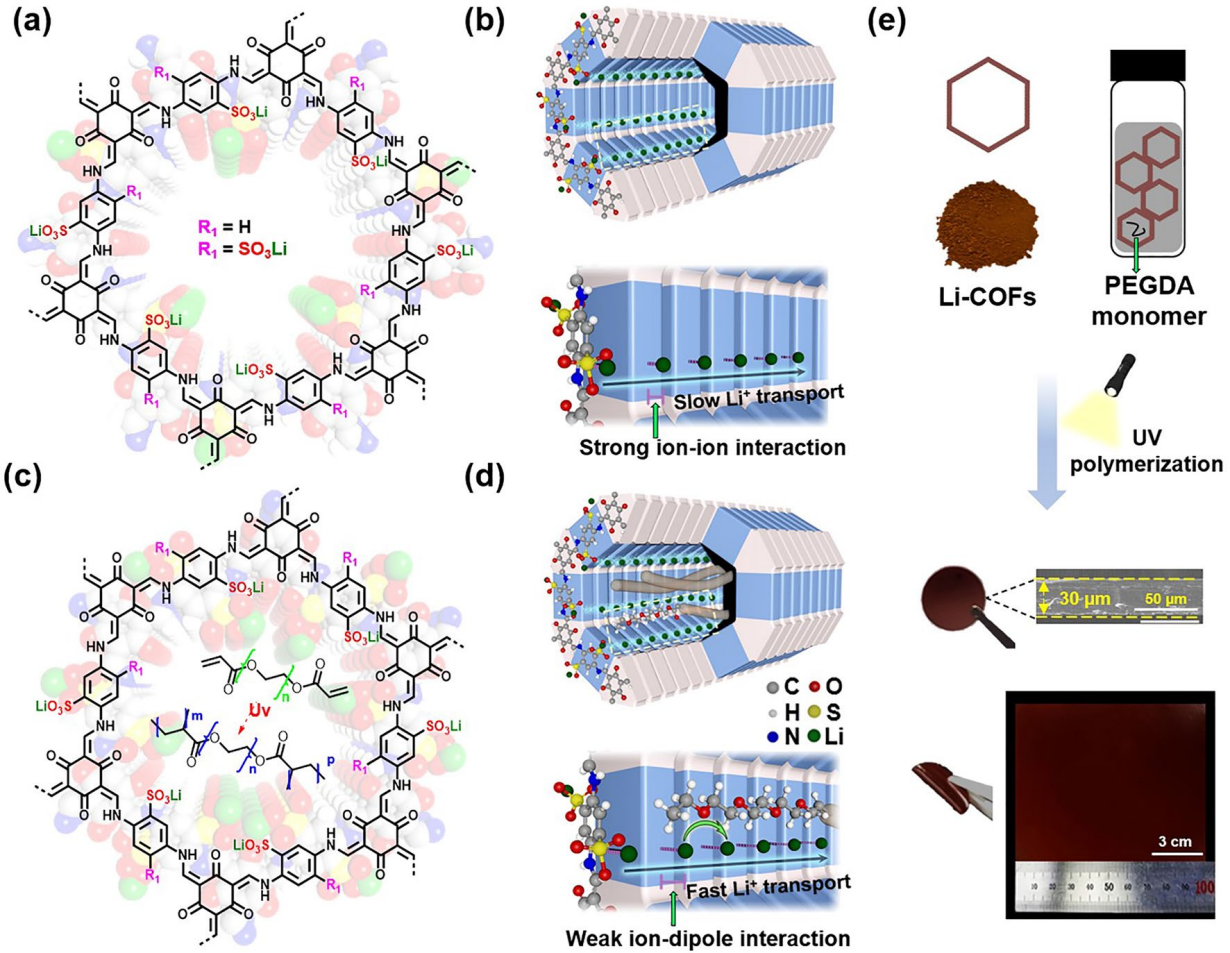
Chemical structure of **a** Li-COF and **b** Li-COF@P_X%_ and conceptual design of their pore functionalization. Li^+^ transport mechanism through the PEGDA-embedded 1D channels in **c** Li-COF and **d** Li-COF@P_X%_. **e** Schematic illustration of the fabrication process of Li-COF@P_X%_ as a thin film, in which its photograph and cross-sectional scanning electron microscopy (SEM) image are shown

Here, we report a new COF strategy based on weak ion–dipole interaction as opposed to traditional strong ion–ion interaction. This chemistry design enables a class of solvent-free COF single-ion conductors (denoted as Li-COF@P_X%_, where X represents pore volume utilization, Fig. 1a, b and Figs. S1, S2) that outperform previously reported COF single-ion conductors. The ion–dipole interaction in the Li-COF@P_X%_ is regulated by embedding polyethylene glycol diacrylate (PEGDA) in the COF pores. The oxygen (O) atoms of carbonyl groups in the embedded PEGDA allowed an ion–dipole interaction with Li^+^ (from the COF). Considering that the ion–dipole interaction is weaker than the ion-ion interaction [37], we suggest that the intermolecular interaction of Li^+^ (ion) with PEGDA (dipole) in the Li-COF@P_X%_ could be weaker than those of traditional single-ion conductors with negatively charged moieties (Nafion with sulfonates, garnet with oxygen sublattices, and others), eventually facilitating the ion dissociation and Li^+^ migration. Consequently, the Li-COF@P_X%_ enabled facile Li^+^ conduction through the PEGDA-embedded 1D channels (Fig. 1c, d). Particularly, the Li-COF-2@P_75%_ exhibited high Li^+^ conductivity (*σ*_Li_^+^  = 8.9 × 10^–5^ S cm^–1^) and Li^+^ transference number (*t*_Li_^+^  = 0.95), as well as a low activation energy forion conduction (*E*_a_ = 0.11 eV), which exceeds those of previously reported solid organic single-ion conductors based on strong ion–ion interaction. In addition, the PEGDA embedded in the COF allowed the formation of a self-standing flexible single-ion conductor film (Fig. 1e). To explore the practical application for all-solid-state Li batteries, the Li-COF-2@P_75%_ was assembled with a Li-metal anode and a 5,5’-dimethyl-2,2’-bis-p-benzoquinone (Me_2_BBQ) cathode (selected as a model organic electrode owing to its high specific capacity and low cost; however, it dissolves in liquid electrolytes [38]). The resultant all-solid-state Li organic batteries (ASSLOBs) exhibited high specific capacity (~ 300 mAh g_Me2BBQ_^−1^) and long cycle retention (88.3% after 2000 cycles) under ambient operating conditions, which outperforms those of previously reported ASSLOBs. This result demonstrates that the intrinsic challenge related to the dissolution of organic electrode materials upon contact with liquid electrolytes can be resolved by the Li-COF-2@P_75%_, highlighting its electrochemical viability as a promising solid and mechanically compliant single-ion conductor platform for ASSLOBs.

## Experimental Section

### Materials

1,4-phenylenediamine-2-sulfonic acid, 1,4-phenylenediamine-2,5-sulfonic acid, 1,3,5-triformylphloroglucinol, poly(ethylene glycol) diacrylate, 1,4-dioxane, mesitylene, acetic acid (HOAc), and other chemicals were purchased from Sigma Aldrich, Tokyo Chemical Industry Co., Ltd, DAEJUNG Co., Ltd, and Yanshen Technology Co., Ltd.

### Preparation of Li-COFs and Li-COF@PX_%_

#### Synthesis of Li-COF-1

2,4,6-Triformylphloroglucinol (63.0 mg, 0.3 mmol), 2,5-diaminobenzenesulfonic acid (84.7 mg, 0.45 mmol), 1,4-dioxane (1.2 mL), 1,3,5-trimethylbenzene (0.8 mL), and acetic acid (6 M 0.6 mL) were added into a Pyrex tube. Thereafter, the mixture was flash-frozen under liquid nitrogen and degassed through three freeze–pump–thaw cycles. Thereafter, the tube was sealed and heated at 120 °C for 3 days, after which the resulting precipitate was collected by filtration and washed with dimethylacetamide and acetone. The obtained product was extracted using Soxhlet extraction with tetrahydrofuran for 12 h and dried under vacuum at 120 ºC overnight (yield: 119.7 mg, 91%). The as-synthesized SO_3_H-COF-1 (200 mg) was suspended in lithium acetate solution (5 M, 20 mL) and stirred for 3 days at room temperature. The resulting powders were collected by filtration and washed with deionized water, and this experiment was performed for three times. Lastly, the Li-COF-1 was washed three times with deionized water (50 mL) and acetone (10 mL), and subjected to vacuum drying at 120 °C overnight to obtain the Li-COF-1 powders (yield: 173.5 mg).

#### Synthesis of Li-COF-2

2,4,6-Triformylphloroglucinol (63.0 mg, 0.3 mmol), 2,5-diaminobenzene-1,4-disulfonic acid (120.6 mg, 0.45 mmol), 1,4-dioxane (1.2 mL), 1,3,5-trimethylbenzene (0.8 mL), and acetic acid (6 M, 0.6 mL) were added into a Pyrex tube, after which the mixture was flash-frozen under liquid nitrogen and degassed through three freeze–pump–thaw cycles. Thereafter, the tube was sealed and heated at 120 °C for 3 days, and the resulting precipitate was collected by filtration and washed with dimethylacetamide and acetone. Subsequently, the product was extracted using Soxhlet extraction with tetrahydrofuran for 12 h and dried under vacuum at 120 °C overnight (yield: 147.3 mg, 81%). The obtained SO_3_H-COF-2 (200 mg) was suspended in lithium acetate solution (5 M, 40 mL) and stirred for 3 days at room temperature, after which the resulting powders were collected by filtration and washed with deionized water, and this experiment was performed three times. Lastly, the Li-COF-2 was washed three times with deionized water (50 mL) and acetone (10 mL), after which it was subjected to vacuum drying at 120 °C overnight to obtain the Li-COF-2 powders (yield: 169.2 mg).

#### ***Synthesis of Li-COF@P***_***X%.***_

The Li-COF was added to a mixture of poly(ethylene glycol) diacrylate (PEGDA, Mn = 250) to prepare mixtures (Li-COF/PEGDA monomer (with 5 wt% 2-hydroxy-2-methylpropiophenone (HMPP) as a photoinitiator)). The obtained mixtures (Li-COF/PEGDA) were subjected to ultrasonication (for 2 h) followed by ball milling (for 0.5 h) to achieve a good dispersion state. The infiltration of the PEGDA into the pores of Li-COF was performed using a low pressure-driven method. Specifically, a predetermined amount of PEGDA monomer was loaded into the degassed Li-COF, after which the sample was subjected to vacuum treatment (0.5 kPa) for 2 h to enable the infiltration of PEGDA monomer into the pores of the crystalline COF. The mixtures were then exposed to UV irradiation (performed using a Hg UV-lamp (Lichtzen) with an irradiation peak intensity of approximately 2000 mW cm^−2^) for less than 1 min to allow the crosslinking of PEGDA monomer, followed by thermal annealing at 80 °C to obtain the Li-COF@P_X%_ Thereafter, the sample was punched into discs (Φ = 13 mm). The dried thin film was pressed into a solid electrolyte film using a uniaxial hydraulic press (Hefei Kejing Materials Technology Co., Ltd.) at a pressure of 220 MPa at 120 °C for 1 h. The maximum PEGDA content in the Li-COF-1@P_100%_ calculated using the material information (density of PEGDA (1.12 g cm^−3^) and pore volume of Li-COF-1 (0.29 cm^3^ g^−1^) was 32%. The Li-COFs@P_X%_ samples were synthesized using the same process except the different loading amounts of PEGDA polymer.

## Results and Discussion

### Structural Characterizations

Li-COF-1 and Li-COF-2 were fabricated using a two-step synthesis procedure, which is schematically illustrated in (Figs. S1–S2). A major difference in the chemical structure of Li-COF-1 and Li-COF-2 is the number of Li^+^. The Li-COF-2 was designed to have twice the number of Li^+^ compared to that of Li-COF-1. To synthesize Li-COF-1 and Li-COF-2, first, SO_3_HCOF-1 and SO_3_H-COF-2 were synthesized using a solvothermal reaction. Thereafter, Li-COF-1 and Li-COF-2 were prepared via a cation exchange reaction between the obtained SO_3_H-COF and Li acetate for three times. The Li-COF-1 and Li-COF-2 were characterized using Fourier transform infrared.

(FT-IR) spectroscopy, ^13^Carbon magic angle spin solid-state nuclear magnetic resonance (NMR) spectroscopy, powder X-ray diffraction (PXRD), field emission scanning electron microscopy (FE-SEM), and energy dispersive X-ray spectroscopy (EDS) mapping analyses (Figs. S3–S7).

The porosities of the Li-COF-1 and Li-COF-2 were measured using nitrogen sorption isotherms at 77 K (Fig. S8a, b). The Brunauer–Emmett–Teller (BET) surface areas of Li-COF-1 and Li-COF-2 were 343 and 95 m^2^ g^−1^, respectively, and their pore volumes were 0.29 and 0.21 cm^3^ g^−1^, respectively. The pore size distributions of Li-COF-1 and Li-COF-2 were centered at 1.2 nm (inset of Fig. S8a, b). The crystalline structures of Li-COF-1 and Li-COF-2 were confirmed using PXRD analysis. A prominent signal was observed in the PXRD pattern of Li-COF-1 at 4.60°, and other weak peaks were observed at 7.78°, 14.08°, and 26.42° (Fig. S9a, red), which were assigned to the (100), (110), (020), and (001) diffractions, respectively. Similarly, the diffraction peaks of Li-COF-2 were observed at 4.72°, 7.80°, 14.22°, and 26.52° (Fig. S10a, red), corresponding to the (100), (110), (020), and (001) facets, respectively. The experimental PXRD results of Li-COF-1 and Li-COF-2 were in good agreement with the simulated AA stacking patterns (Figs. S9a–S10a, green). In contrast, the simulated AB-staggered mode of Li-COF-1 and Li-COF-2 was inconsistent with the experimental results (Figs. S9a–S10a, purple). Furthermore, a unit cell structure was confirmed for both Li-COF-1 (Fig. S9b) and Li-COF-2 (Fig. S10b).

Next, self-standing pellets of Li-COF (≥ 200 μm) with interparticle voids were prepared using a cold-pressing method. In addition, thin films (thickness ~ 30 μm) of Li-COF@P_X%_ were prepared without using processing solvents as follows: the PEGDA monomer was embedded into the pores of the degassed Li-COF by vacuum-assisted infiltration [29]. The resulting Li-COF was then exposed to UV irradiation, followed by thermal annealing at 80 ℃ to obtain the Li-COF@P_X%_ (Fig. S11). The dried samples were pressed into a Li-COF@P_X%_ thin film using a uniaxial hydraulic press at 220 MPa of pressure for 1 h at 120 °C. Based on the material information (density of PEGDA (1.12 g cm^−3^) and pore volume of Li-COF (see also the Methods Details) [25], the calculated maximum PEGDA content in Li-COF-1 and Li-COF-2 was 32% and 24%, respectively. The presence of the elastic P_PEGDA_ endowed the Li-COF-2@P_X%_ with mechanical flexibility and manufacturing scalability owing to the compliant PEGDA in the channel of the Li-COF (Fig. 1e). To obtain detailed information on the structure, porosity, and crystallinity of Li-COFs@P_X%_, the thin films were converted into powders using a simple grinding method. Compared to P_PEGDA_, strong peaks related to CH_2_ bonds (2870 and 1189 cm^−1^) and C–O bonds (1721 cm^−1^) were observed in the FT-IR spectra of Li-COF@P_X%_ powders (Fig. S12). With an increase in the P_PEGDA_ content, the relative intensity of the signals in the FT-IR spectra increased. In addition, a downshift of the C–O bond observed in the PXRD spectra of Li-COFs@P_X%_ powders compared to the pristine P_PEGDA_ exhibited the interactions between the O atoms of P_PEGDA_ and Li-COF. After loading P_PEGDA_, the nitrogen uptake of Li-COF@P_X%_ decreased, confirming the incorporation of P_PEGDA_ into Li-COF@P_100%_. Additionally, the BET surface area of Li-COF@P_100%_ reduced to less than 10 m^2^ g^−1^ and the pore volume was less than 0.01 cm^3^ g^−1^ (Fig. S13). The PXRD patterns of the Li-COFs@P_X%_ powders after the impregnation with P_PEGDA_ showed high crystallinity (Fig. S14), indicating that the Li-COFs@P_X%_ samples were successfully synthesized.

### Electrochemical Properties and Mechanism

**Fig. 2:**
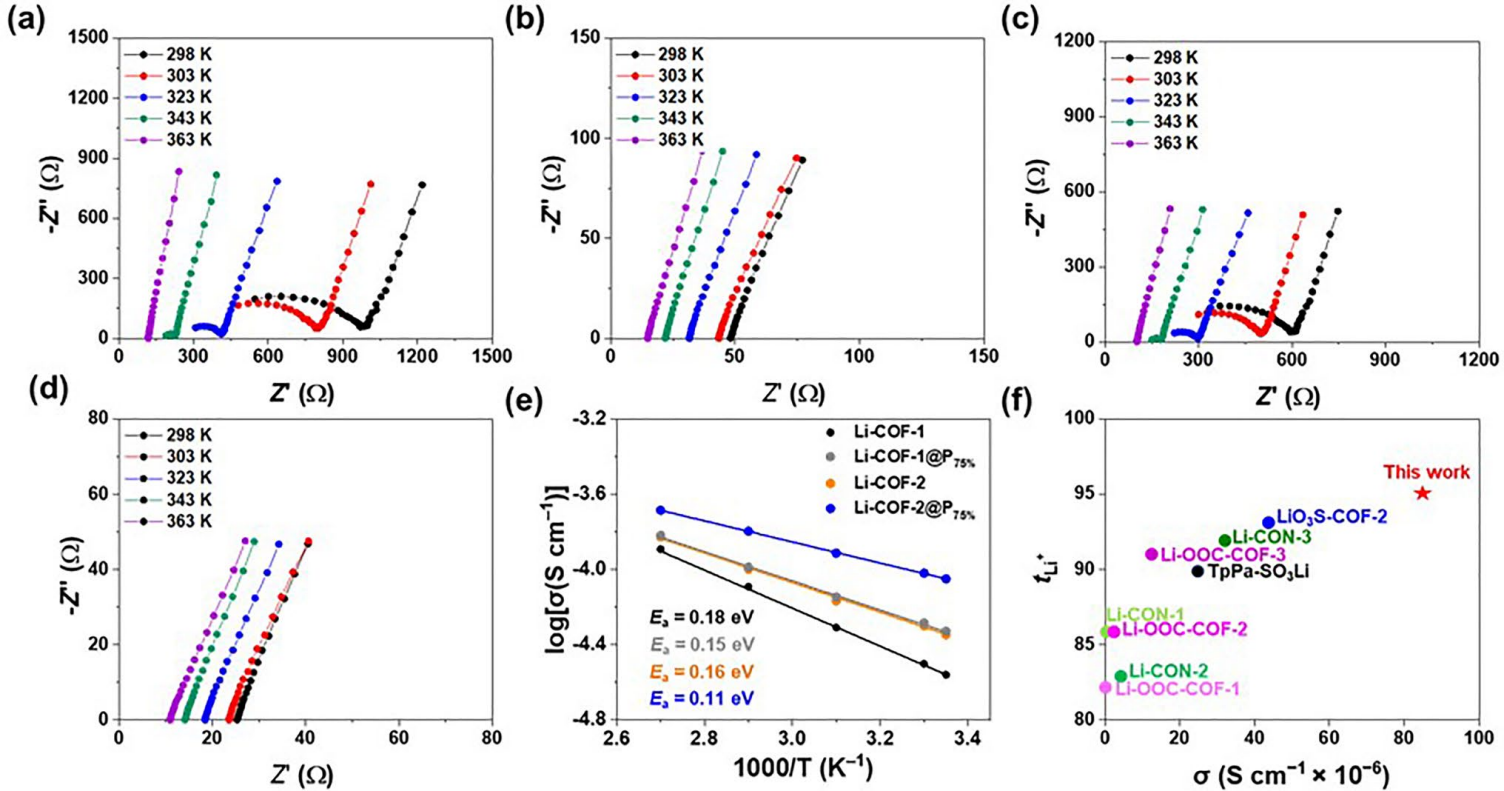
Electrochemical impedance spectroscopy (EIS) profiles of **a** Li-COF-1, **b** Li-COF-1@P_75%_, **c** Li-COF-2, and **d** Li-COF-2@P_75%_ measured at different temperatures (from 298 to 363 K). **e** Arrhenius plots for the ionic conductivity of Li-COF-1, Li-COF-1@P_75%_, Li-COF-2, and Li-COF-2@P_75%_. **f** Comparison of the Li^+^ conductivity of Li-COF-2@P_75%_ to those of the previously reported single-ion conducting COFs

The ionic conductivity of the fabricated Li-COF and Li-COF@P_X%_ was evaluated at different temperatures (from 298 to 363 K) using electrochemical impedance spectroscopy (EIS) analysis (Fig. 2a–e and Figs. S15, S16). In the absence of additional Li salts or organic solvents, the Li-COF-1 exhibited an ionic conductivity of 2.7 × 10^−5^ S cm^−1^ at room temperature (Fig. 2a). After the incorporation of P_PEGDA_ into Li-COF-1, the ionic conductivities of Li-COF-1@P_25%_, Li-COF-1@P_50%_, and Li-COF-1@P_75%_ increased to 3.6 × 10^−5^, 4.1 × 10^−5^, and 5.1 × 10^−5^ S cm^−1^, respectively (Fig. 2b, Fig. S15, and Table S1). After incorporation into the pore channel, the oxygen-rich groups of P_PEGDA_ enhanced Li^+^ transport owing to the weak ion (Li^+^)–dipole interactions, thereby facilitating the dissociation of the ion − counter anion (mobile Li^+^ and anionic channels) interaction and fast Li^+^ migration in the anionic nanochannel. However, when the P_PEGDA_ content was higher than the pore volume of Li-COF-1, the ionic conductivities of Li-COF@P_100%_ and Li-COF@P_125%_ slightly decreased to 2.3 × 10^−5^ and 1.8 × 10^−5^ S cm^−1^, respectively (Fig. S15 and Table S1). This decrease in the ionic conductivity was attributed to the decrease in the available Li^+^ content caused by the relatively larger amount of the PEGDA (Table S1). Under optimal conditions, a similar phenomenon was observed in the Li-COF-2 and Li-COF-2@P_X%_, and the highest ionic conductivity (8.9 × 10^−5^ S cm^−1^) was achieved by Li-COF-2@P_75%_ (Fig. 2d), which was almost twice that of Li-COF-2 (4.9 × 10^−5^ S cm^−1^, Fig. 2c). This value outperforms those of previously reported COF-based single-ion conductors and other organic conductors (Fig. 2f and Table S2) [30–33, 39, 40].

The Arrhenius plot shows a proportional increase in the logarithmic ionic conductivity with increasing temperature (Figs. 2a–d, S15 and S16). The *E*_a_ values of Li-COF and Li-COF@P_X%_ were obtained from their Arrhenius plots (Figs. 2e, S17 and S18). The lowest *E*_a_ value (0.11 eV) was observed for Li-COF-2@P_75%_ (Fig. 2e), which is one of the lowest values provided by COF based single-ion conductors and other organic conductors reported to date (Table S1–S3) [30–33, 39, 40].

To demonstrate the single Li^+^ conduction behavior of Li-COF and Li-COF@P_X%_, their *t*_Li_^+^ was examined at 298 K using a potentiostatic polarization method [39–42]. The *t*_Li_^+^ values of Li-COF-1@P_75%_ and Li-COF-2@P_75%_ were 0.93 and 0.95, respectively, which are higher than those of Li-COF-1 and Li-COF-2 (Figs. S19, S20, and Table S1). The *t*_Li_^+^ value of Li-COF-2@P_75%_ is significantly higher than those of the previously reported solid-state porous crystalline ion conductors (Table S2 and Fig. 2f). It should be noted that the Li^+^ conductance, rather than the Li^+^ conductivity, has a more significant influence on the electrochemical performance of all-solid-state Li batteries. Compared to the thick (200 μm) Li-COF-2 (2.9 mS), the thin (30 μm) Li-COF-2@P_75%_ exhibited ionic conductance (39.5 mS, in Fig. S21). Furthermore, the ionic conductance of the thin Li-COF-2@P_75%_ was higher than that of previously reported 700 μm-thick inorganic Li_6_PS_5_Cl_0.5_Br_0.5_ pellet (29 mS) [43].

**Fig. 3:**
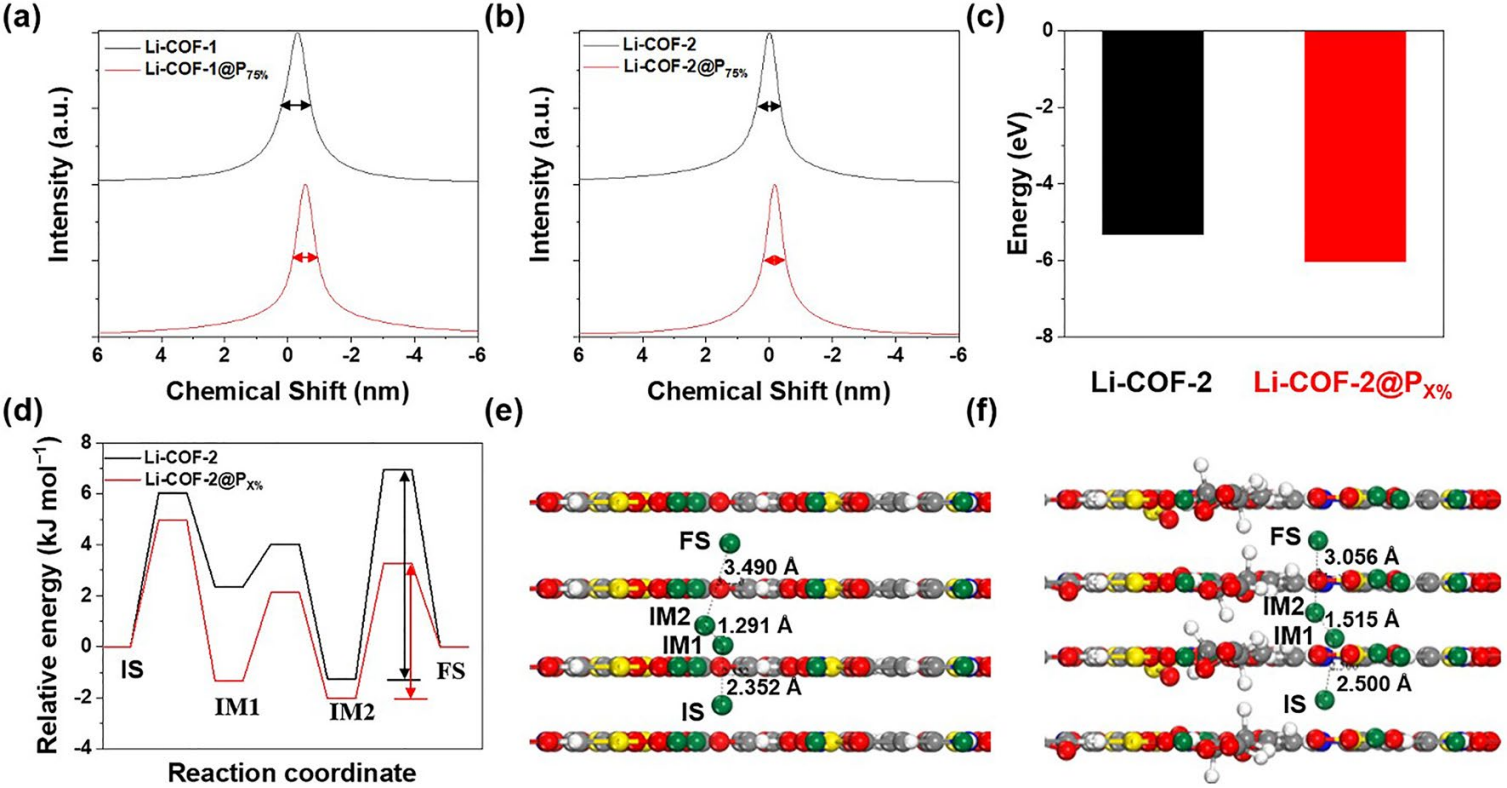
^7^Li MAS NMR spectra of **a** Li-COF and **b** Li-COF@P_75%_. **c** Dissociation energy of Li-COF-2 and Li-COF-2@P_X%_. **d** Theoretical elucidation of Li^+^ migration behavior within the pore with corresponding energy diagrams. Theoretical elucidation of the Li^+^ migration behavior of **e** Li-COF-2 and **f** Li-COF-2@P_X%_ (The initial, intermediate, and final states are abbreviated as IS, IM, and FS, respectively)

The local chemical environment and molecular dynamics of Li^+^ in Li-COF and Li-COF@P_75%_ were investigated using solid state ^7^Li NMR (Fig. 3a, b). A broad signal was observed in the ^7^Li NMR spectra of Li-COF-1 and Li-COF-2, indicating the sluggish Li^+^ conduction in the pores of Li-COF. In contrast, Li-COF-1@P_75%_ and Li-COF-2@P_75%_ exhibited a narrower width [30, 42, 43] and an upfield shift [44, 45] in the ^7^Li spectra, indicating the prevalence of freely mobile Li^+^. In addition, the shift in the spectra of the Li-COF-1@P_75%_ and Li-COF-2@P_75%_ was more pronounced than those of Li-COF-1 and Li-COF-2. This difference in the chemical shift was attributed to the weak ion–dipole interaction between Li^+^ and the oxygen of the PEGDA. The weak ion–dipole interaction contributed to the enhancement of Li^+^ migration in the anionic channel of COFs.

The Li^+^ transport phenomena in the Li-COF-2 and Li-COF-2@P_X%_ were theoretically elucidated by conducting density functional theory (DFT) calculations. The perpendicular pathway is an effective route for Li^+^ transport to achieve the lower migration barriers (*E*_m_) [30, 33, 45]. Next, the dissociation energy and migration barriers of the Li-COF-2 model were investigated. After embedding the PEGDA in the COF pores, the dissociation energy of Li sulfonate decreased from – 5.32 to – 6.03 eV (Figs. 3c and S22), indicating that the oxygen atoms were beneficial in promoting Li dissociation via ion–dipole interaction. In addition, the initial, intermediate, and final states (IS, IM1, IM2, and FS) of Li^+^ were investigated (Fig. 3d–f). The results revealed that Li-COF-2 exhibited a high *E*_m_ of 8.22 kcal mol^−1^ in the initial state, whereas Li-COF-2@P_X%_ showed a lower *E*_m_ (5.31 kcal mol^−1^). When the PEGDA was fused into the anionic channel of the Li-COF, the Li^+^ migration barriers were lowered, resulting in fast Li^+^ transport.

**Fig. 4:**
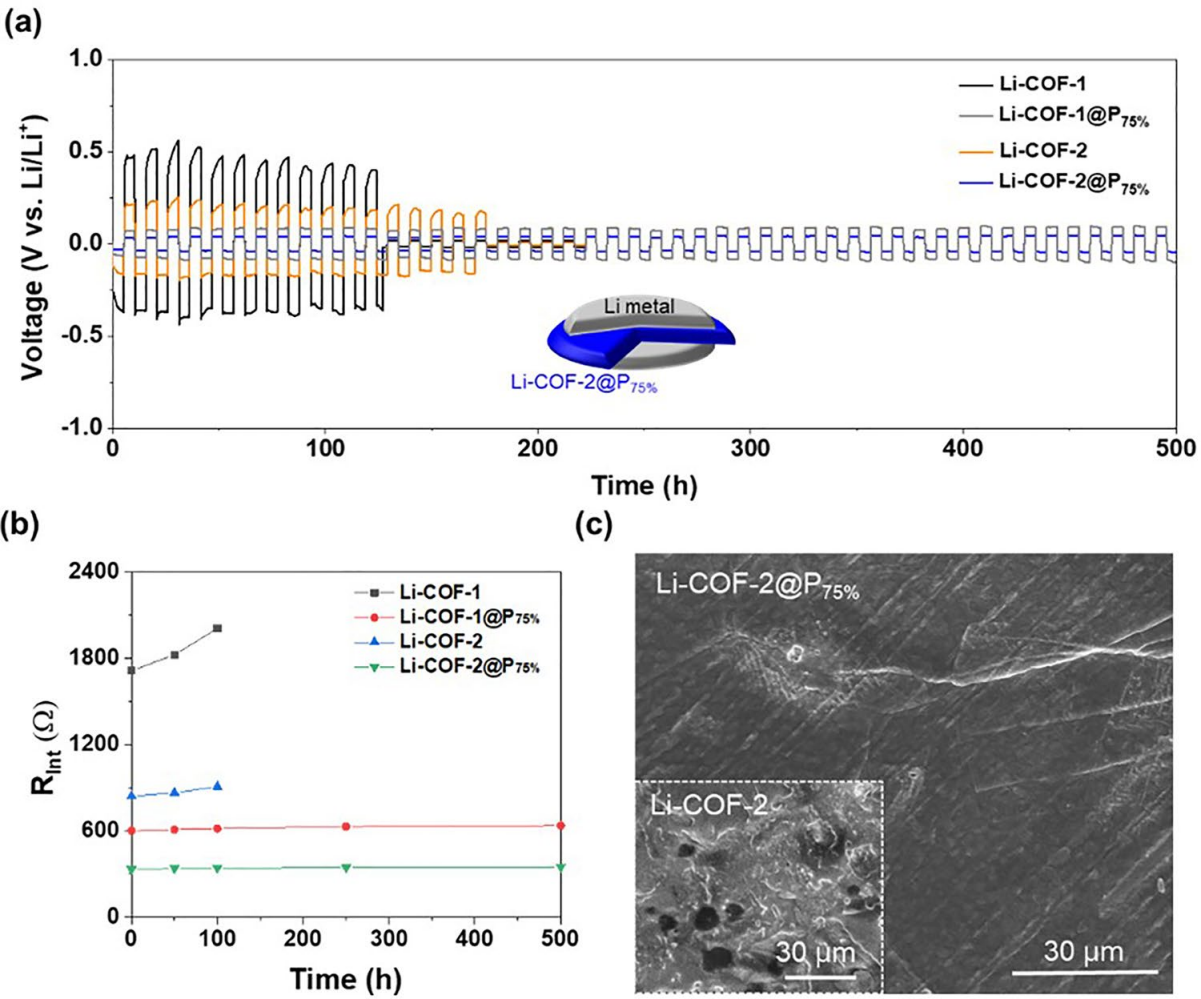
Electrochemical compatibility with Li-metal anodes. **a** Galvanostatic Li plating/stripping profile of the Li||Li symmetric cell containing Li-COF-1, Li-COF-1@P_75%_, Li-COF-2, and Li-COF-2@P_75%_ at a current density of 0.05 mA cm^−2^ and areal capacity of 0.25 mAh cm^−2^. **b** Change in the *R*_Int_ of the cell during the cycling test. **c** FE-SEM images of the Li-metal anode surface of Li-COF-2@P_75%_ and Li-COF-2 after the cycling test (100 h)

The applicability of Li-COF and Li-COF@P_75%_ as a new solid-state electrolyte for Li-metal anodes was investigated using the Li||Li symmetric cell configuration (inset of Fig. 4a). Galvanostatic Li plating/stripping on the Li-metal anodes was performed repeatedly at a current density of 0.05 mA cm^−2^ for 5 h per cycle. The symmetric cell of Li-COF@P_75%_ exhibited stable and reliable Li plating/stripping behavior for over 500 h without any significant increase and an irreversible fluctuation in the overpotential compared to that of the Li-COF (Fig. 3a). This superior cyclability was verified by monitoring the change in the interfacial resistance (*R*_Int_) of the cell as a function of the cycling time (Fig. 4b and Table S4). The increase in *R*_Int_ was retarded during the cycling, indicating the good interfacial stability of Li-COF-2@P_75%_ with Li-metal anodes. This result was confirmed by the clean and smooth surface of the Li-metal anodes after the cycling test (Fig. 4c). Additionally, random Li deposition was hardly observed, indicating that the Li-COF-2@P_75%_ enabled uniform Li^+^ flux to the Li-metal anodes. In addition, the PXRD analysis revealed that the ordered structure of Li-COF-2@P_75%_ was not disrupted after the cycling test (Fig. S23). These results demonstrate the promising potential of Li-COF-2@P_75%_ as a solvent-free, organic single Li^+^ conductor, which enables stable electrochemical compatibility with the Li metal anodes.

**Fig. 5:**
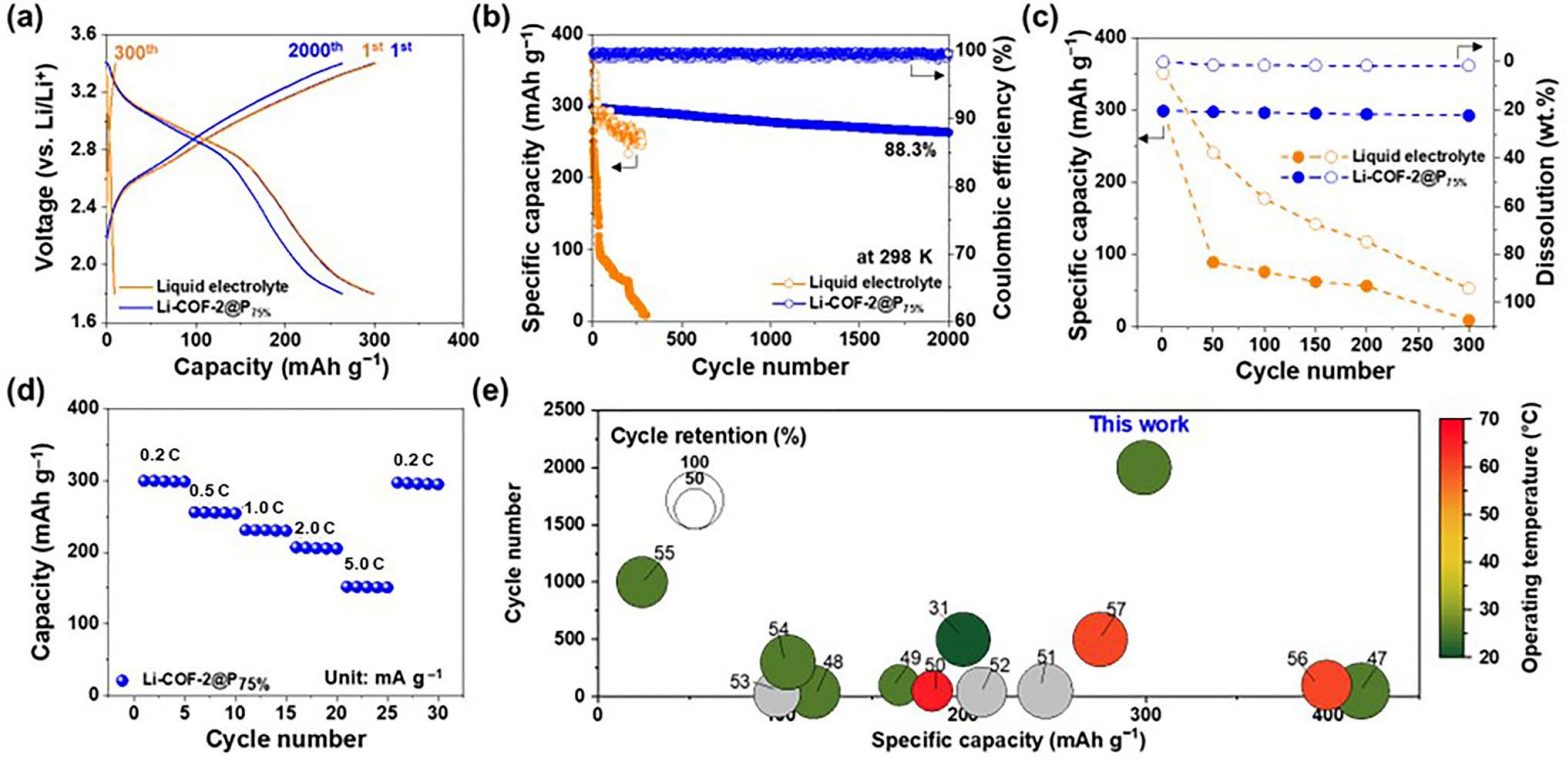
Electrochemical performance of the ASSLOBs. **a** Voltage profiles and **b** cycling performance of the ASSLOBs (Me_2_BBQ‖Li assembled with the Li-COF-2@P_75%_ (vs. liquid electrolyte) at a charge/discharge current density of 0.2/0.2 C and voltage range of 1.8–3.4 V at 298 K. **c** Specific capacity and dissolution of the Me_2_BBQ cathode as a function of cycle number (Li-COF-2@P_75%_ vs. liquid electrolyte). **d** Rate capability of the ASSLOBs with the Li-COF-2@P_75%_, in which the discharge current densities were varied from 0.2 to 5.0 C at a fixed charge current density of 0.2 C. **e** Comparison of the Li-COF-2@P_75%_ and the previously reported organic electrolytes in terms of specific capacity of organic cathode materials (x-axis), cycle number (y-axis), operating temperature (heatmap), and cycle retention (diameter). The detailed values assigned to each circle were described in Table S5

The Li-COF-2@P_75%_ was combined with a Li-metal anode and a Me_2_BBQ cathode to explore its practical application in ASSLOBs. The Me_2_BBQ is known to provide a lower cost and high specific capacity based on a three-electron redox reaction (332 mAh g^−1^), in comparison to conventional metal oxide-based cathode active materials [38]. However, the Me_2_BBQ suffers from undesirable dissolution in liquid electrolytes (Fig. S24), resulting in poor cycling performance [39]. We expect that the Li-COF-2@P_75%_ can be proposed as a promising solid Li^+^ conductor to solve to this problem. The ASSLOB assembled with the Me_2_BBQ exhibited a reversible capacity of ~ 300 mAh g^−1^ at the first cycle in the voltage range of 1.8–3.4 V (vs. Li/Li^+^) at room temperature (Fig. 5a).

Notably, the ASSLOB with the Me_2_BBQ showed stable capacity retention with cycling (88.3% after 2000 cycles) whereas the control cell with a liquid electrolyte showed rapid capacity degradation after only 50 cycles (Fig. 5b). This result was verified by examining the relationship between the capacity and Me_2_BBQ dissolution as a function of the cycle number (Figs. 5c and S56). The ASSLOB with the Me_2_BBQ achieved the decent discharge rate capability at various current densities ranging from 0.2 to 5.0 C (Fig. 5d). In addition, the ASSLOB with the Me_2_BBQ still exhibited stable cycling performance (84.2% after 300 cycles) at ambient operating conditions (i.e., room temperature without external pressure) under a high current density of 5.0 C (Fig. S26). The superior electrochemical performance of the ASSLOB (this study) over the previously reported ASSLOBs was highlighted through a comparative analysis encompassing the specific capacity of organic cathode materials (x-axis), cycle number (y-axis), operating temperature (heatmap), and cycle retention (diameter) (Fig. 5e and Table S5) [46–58]. The significantly improved cyclability was observed at the ASSLOB (this study), whereas most of the previous works on ASSLOBs suffered from poor cycling retention (< 500 cycles) along with fast capacity fading rate due to the dissolution of organic electrode materials into liquid electrolytes. This result demonstrates the viability of Li-COF-2@P_75%_ as a promising solid Li^+^ conductor suitable for high-capacity organic electrode materials.

## Conclusions

In summary, we presented the Li-COF@P as a solvent-free, mechanically compliant organic single-ion conductor based on weak ion–dipole interaction, in contrast to conventional organic single-ion conductors based on strong ion–ion interaction. The weak ion (Li^+^ from the COF)–dipole (oxygen from the PEGDA embedded in the COF pores) interaction promoted the ion dissociation and Li^+^ migration, thereby facilitating Li^+^ conduction through the functionalized 1D channels. The Li-COF-2@P_75%_ exhibited facile Li^+^ conduction behavior in the absence of Li salts and organic solvents, outperforming those of the previously reported solid organic single-ion conductors based on ion–ion interaction. When combined with the Me_2_BBQ cathode, the Li-COF-2@P_75%_ enabled the resulting full cell to achieve a stable cyclability (88.3% after 2000 cycles) under ambient operating conditions. The Li-COF@P strategy based on the ion–dipole interaction holds promise as a new solid electrolyte platform for all-solid-state batteries and opens a new perspective in the design of COF single-ion conductors as a viable alternative to the currently prevalent inorganic solid electrolytes.

## Supplementary Information

Below is the link to the electronic supplementary material.

Supplementary file1 (DOCX 3519 KB)

## References

[1] C. Yang, Z. Suo, Hydrogel ionotronics. Nat. Rev. Mater. **3**, 125–142 (2018). 10.1038/s41578-018-0018-7

[2] H.J. Kim, B. Chen, Z. Suo, R.C. Hayward, Ionoelastomer junctions between polymer networks of fixed anions and cations. Science **367**, 773–776 (2020). 10.1126/science.aay8467 32054759 10.1126/science.aay8467

[3] W. Zhang, D.H. Seo, T. Chen, L. Wu, M. Topsakal et al., Kinetic pathways of ionic transport in fast-charging lithium titanate. Science **367**, 1030–1034 (2020). 10.1126/science.aax3520 32108110 10.1126/science.aax3520

[4] C.S. Rustomji, Y. Yang, T.K. Kim, J. Mac, Y.J. Kim et al., Liquefied gas electrolytes for electrochemical energy storage devices. Science **356**, al4263 (2017). 10.1126/science.aal4263 10.1126/science.aal4263 28619715

[5] Q. Zhao, S. Stalin, C.-Z. Zhao, L.A. Archer, Designing solid-state electrolytes for safe, energy-dense batteries. Nat. Rev. Mater. **5**, 229–252 (2020). 10.1038/s41578-019-0165-5

[6] C. Fang, J. Li, M. Zhang, Y. Zhang, F. Yang et al., Quantifying inactive lithium in lithium metal batteries. Nature **572**, 511–515 (2019). 10.1038/s41586-019-1481-z 31435056 10.1038/s41586-019-1481-z

[7] J. Zheng, Q. Zhao, T. Tang, J. Yin, C.D. Quilty et al., Reversible epitaxial electrodeposition of metals in battery anodes. Science **366**, 645–648 (2019). 10.1126/science.aax6873 31672899 10.1126/science.aax6873

[8] J. Janek, W.G. Zeier, A solid future for battery development. Nat. Energy **1**, 16141 (2016). 10.1038/nenergy.2016.141

[9] M. Winter, B. Barnett, K. Xu, Before Li ion batteries. Chem. Rev. **118**, 11433–11456 (2018). 10.1021/acs.chemrev.8b00422 30500179 10.1021/acs.chemrev.8b00422

[10] Y. An, X. Han, Y. Liu, A. Azhar, J. Na et al., Progress in solid polymer electrolytes for lithium-ion batteries and beyond. Small **18**, e2103617 (2022). 10.1002/smll.202103617 34585510 10.1002/smll.202103617

[11] T. Zhou, X. Huang, N. Ding, Z. Lin, Y. Yao et al., Porous polyelectrolyte frameworks: synthesis, post-ionization and advanced applications. Chem. Soc. Rev. **51**, 237–267 (2022). 10.1039/d1cs00889g 34877581 10.1039/d1cs00889g

[12] D. Luo, M. Li, Q. Ma, G. Wen, H. Dou et al., Porous organic polymers for Li-chemistry-based batteries: functionalities and characterization studies. Chem. Soc. Rev. **51**, 2917–2938 (2022). 10.1039/d1cs01014j 35285470 10.1039/d1cs01014j

[13] P.J. Waller, F. Gándara, O.M. Yaghi, Chemistry of covalent organic frameworks. Acc. Chem. Res. **48**, 3053–3063 (2015). 10.1021/acs.accounts.5b00369 26580002 10.1021/acs.accounts.5b00369

[14] J. Li, X. Jing, Q. Li, S. Li, X. Gao et al., Bulk COFs and COF nanosheets for electrochemical energy storage and conversion. Chem. Soc. Rev. **49**, 3565–3604 (2020). 10.1039/d0cs00017e 32369058 10.1039/d0cs00017e

[15] S.-Y. Ding, W. Wang, Covalent organic frameworks (COFs): from design to applications. Chem. Soc. Rev. **42**, 548–568 (2013). 10.1039/c2cs35072f 23060270 10.1039/c2cs35072f

[16] D. Zhu, G. Xu, M. Barnes, Y. Li, C.-P. Tseng et al., Covalent organic frameworks for batteries. Adv. Funct. Mater. **31**, 2100505 (2021). 10.1002/adfm.202100505

[17] H. Wang, Z. Zeng, P. Xu, L. Li, G. Zeng et al., Recent progress in covalent organic framework thin films: fabrications, applications and perspectives. Chem. Soc. Rev. **48**, 488–516 (2019). 10.1039/c8cs00376a 30565610 10.1039/c8cs00376a

[18] R.-R. Liang, S.-Y. Jiang, A. Ru-Han, X. Zhao, Two-dimensional covalent organic frameworks with hierarchical porosity. Chem. Soc. Rev. **49**, 3920–3951 (2020). 10.1039/d0cs00049c 32427238 10.1039/d0cs00049c

[19] F. Meng, S. Bi, Z. Sun, B. Jiang, D. Wu et al., Synthesis of ionic vinylene-linked covalent organic frameworks through quaternization-activated Knoevenagel condensation. Angew. Chem. Int. Ed. Engl. **60**, 13614–13620 (2021). 10.1002/anie.202104375 33844881 10.1002/anie.202104375

[20] D.A. Vazquez-Molina, G.S. Mohammad-Pour, C. Lee, M.W. Logan, X. Duan et al., Mechanically shaped two-dimensional covalent organic frameworks reveal crystallographic alignment and fast Li-ion conductivity. J. Am. Chem. Soc. **138**, 9767–9770 (2016). 10.1021/jacs.6b05568 27414065 10.1021/jacs.6b05568

[21] C. Li, D.-D. Wang, G.S.H. Poon Ho, Z. Zhang, J. Huang et al., Anthraquinone-based silicate covalent organic frameworks as solid electrolyte interphase for high-performance lithium–metal batteries. J. Am. Chem. Soc **145**, 24603–24614 (2023). 10.1021/jacs.3c06723 10.1021/jacs.3c06723 37916601

[22] Z. Meng, R.M. Stolz, K.A. Mirica, Two-dimensional chemiresistive covalent organic framework with high intrinsic conductivity. J. Am. Chem. Soc. **141**, 11929–11937 (2019). 10.1021/jacs.9b03441 31241936 10.1021/jacs.9b03441

[23] Y. Hu, N. Dunlap, S. Wan, S. Lu, S. Huang et al., Crystalline lithium imidazolate covalent organic frameworks with high Li-ion conductivity. J. Am. Chem. Soc. **141**, 7518–7525 (2019). 10.1021/jacs.9b02448 30986353 10.1021/jacs.9b02448

[24] G. Zhang, Y.-L. Hong, Y. Nishiyama, S. Bai, S. Kitagawa et al., Accumulation of glassy poly(ethylene oxide) anchored in a covalent organic framework as a solid-state Li^+^ electrolyte. J. Am. Chem. Soc. **141**, 1227–1234 (2019). 10.1021/jacs.8b07670 30576136 10.1021/jacs.8b07670

[25] H. Xu, S. Tao, D. Jiang, Proton conduction in crystalline and porous covalent organic frameworks. Nat. Mater. **15**, 722–726 (2016). 10.1038/nmat4611 27043780 10.1038/nmat4611

[26] L. Yao, C. Ma, L. Sun, D. Zhang, Y. Chen et al., Highly crystalline polyimide covalent organic framework as dual-active-center cathode for high-performance lithium-ion batteries. J. Am. Chem. Soc. **144**, 23534–23542 (2022). 10.1021/jacs.2c10534 36512747 10.1021/jacs.2c10534

[27] S. Xu, M. Richter, X. Feng, Vinylene-linked two-dimensional covalent organic frameworks: synthesis and functions. Acc. Mater. Res. **2**, 252–265 (2021). 10.1021/accountsmr.1c00017

[28] W. Gong, Y. Ouyang, S. Guo, Y. Xiao, Q. Zeng et al., Covalent organic framework with multi-cationic molecular chains for gate mechanism controlled superionic conduction in all-solid-state batteries. Angew. Chem. Int. Ed. **62**, e202302505 (2023). 10.1002/anie.202302505 10.1002/anie.202302505 36992624

[29] D. Guo, D.B. Shinde, W. Shin, E. Abou-Hamad, A.-H. Emwas et al., Foldable solid-state batteries enabled by electrolyte mediation in covalent organic frameworks. Adv. Mater. **34**, e2201410 (2022). 10.1002/adma.202201410 35332970 10.1002/adma.202201410

[30] K. Jeong, S. Park, G.Y. Jung, S.H. Kim, Y.-H. Lee et al., Solvent-free, single lithium-ion conducting covalent organic frameworks. J. Am. Chem. Soc. **141**, 5880–5885 (2019). 10.1021/jacs.9b00543 30888813 10.1021/jacs.9b00543

[31] X. Li, Q. Hou, W. Huang, H.-S. Xu, X. Wang et al., Solution-processable covalent organic framework electrolytes for all-solid-state Li–organic batteries. ACS Energy Lett. **5**, 3498–3506 (2020). 10.1021/acsenergylett.0c01889

[32] G. Zhao, Z. Mei, L. Duan, Q. An, Y. Yang et al., COF-based single Li^+^ solid electrolyte accelerates the ion diffusion and restrains dendrite growth in quasi-solid-state organic batteries. Carbon Energy **5**, e248 (2023). 10.1002/cey2.248

[33] X. Li, K.P. Loh, Recent progress in covalent organic frameworks as solid-state ion conductors. ACS Mater. Lett. **1**, 327–335 (2019). 10.1021/acsmaterialslett.9b00185

[34] S. Yuan, X. Li, J. Zhu, G. Zhang, P. Van Puyvelde et al., Covalent organic frameworks for membrane separation. Chem. Soc. Rev. **48**, 2665–2681 (2019). 10.1039/c8cs00919h 31025660 10.1039/c8cs00919h

[35] H.S. Sasmal, H.B. Aiyappa, S.N. Bhange, S. Karak, A. Halder et al., Superprotonic conductivity in flexible porous covalent organic framework membranes. Angew. Chem. Int. Ed. **57**, 10894–10898 (2018). 10.1002/anie.201804753 10.1002/anie.201804753 29958331

[36] M.C. Senarathna, H. Li, S.D. Perera, J. Torres-Correas, S.D. Diwakara et al., Highly flexible dielectric films from solution processable covalent organic frameworks. Angew. Chem. Int. Ed. **62**, e202312617 (2023). 10.1002/anie.202312617 10.1002/anie.202312617 37851585

[37] F. Biedermann, H.-J. Schneider, Experimental binding energies in supramolecular complexes. Chem. Rev. **116**, 5216–5300 (2016). 10.1021/acs.chemrev.5b00583 27136957 10.1021/acs.chemrev.5b00583

[38] S. Bai, B. Kim, C. Kim, O. Tamwattana, H. Park et al., Permselective metal-organic framework gel membrane enables long-life cycling of rechargeable organic batteries. Nat. Nanotechnol. **16**, 77–84 (2021). 10.1038/s41565-020-00788-x 33139935 10.1038/s41565-020-00788-x

[39] R. Bouchet, S. Maria, R. Meziane, A. Aboulaich, L. Lienafa et al., Single-ion BAB triblock copolymers as highly efficient electrolytes for lithium-metal batteries. Nat. Mater. **12**, 452–457 (2013). 10.1038/nmat3602 23542871 10.1038/nmat3602

[40] K. Jeong, S. Park, S.-Y., Lee Revisiting polymeric single lithium-ion conductors as an organic route for all-solid-state lithium ion and metal batteries. J. Mater. Chem. A **7**, 1917–1935 (2019). 10.1039/C8TA09056D

[41] K.-S. Oh, S. Park, J.-S. Kim, Y. Yao, J.-H. Kim et al., Electrostatic covalent organic frameworks as on-demand molecular traps for high-energy Li metal battery electrodes. ACS Energy Lett. **8**, 2463–2474 (2023). 10.1021/acsenergylett.3c00600

[42] K.-S. Oh, J.-H. Kim, S.-H. Kim, D. Oh, S.-P. Han et al., Single-ion conducting soft electrolytes for semi-solid lithium metal batteries enabling cell fabrication and operation under ambient conditions. Adv. Energy Mater. **11**, 2170151 (2021). 10.1002/aenm.202170151

[43] D.H. Kim, Y.-H. Lee, Y.B. Song, H. Kwak, S.-Y. Lee et al., Thin and flexible solid electrolyte membranes with ultrahigh thermal stability derived from solution-processable Li argyrodites for all-solid-state Li-ion batteries. ACS Energy Lett. **5**, 718–727 (2020). 10.1021/acsenergylett.0c00251

[44] H. Chen, H. Tu, C. Hu, Y. Liu, D. Dong et al., Cationic covalent organic framework nanosheets for fast Li-ion conduction. J. Am. Chem. Soc. **140**, 896–899 (2018). 10.1021/jacs.7b12292 29303576 10.1021/jacs.7b12292

[45] Y. Cao, M. Wang, H. Wang, C. Han, F. Pan et al., Covalent organic framework for rechargeable batteries: mechanisms and properties of ionic conduction. Adv. Energy Mater. **12**, 2200057 (2022). 10.1002/aenm.202200057

[46] Z. Zhu, M. Hong, D. Guo, J. Shi, Z. Tao et al., All-solid-state lithium organic battery with composite polymer electrolyte and pillar[5]quinone cathode. J. Am. Chem. Soc. **136**, 16461–16464 (2014). 10.1021/ja507852t 25383544 10.1021/ja507852t

[47] H. Fei, Y. Liu, Y. An, X. Xu, G. Zeng et al., Stable all-solid-state potassium battery operating at room temperature with a composite polymer electrolyte and a sustainable organic cathode. J. Power. Sources **399**, 294–298 (2018). 10.1016/j.jpowsour.2018.07.124

[48] B. Kim, H. Kang, K. Kim, R.Y. Wang, M.J. Park, All-solid-state lithium-organic batteries comprising single-ion polymer nanoparticle electrolytes. ChemSusChem **13**, 2271–2279 (2020). 10.1002/cssc.202000117 32207562 10.1002/cssc.202000117

[49] W. Li, L. Chen, Y. Sun, C. Wang, Y. Wang et al., All-solid-state secondary lithium battery using solid polymer electrolyte and anthraquinone cathode. Solid State Ion. **300**, 114–119 (2017). 10.1016/j.ssi.2016.12.013

[50] Y. Shi, Y. Chen, Y. Liang, J. Andrews, H. Dong et al., Chemically inert covalently networked triazole-based solid polymer electrolytes for stable all-solid-state lithium batteries. J. Mater. Chem. A **7**, 19691–19695 (2019). 10.1039/C9TA05885K

[51] M. Lécuyer, J. Gaubicher, A.-L. Barrès, F. Dolhem, M. Deschamps et al., A rechargeable lithium/quinone battery using a commercial polymer electrolyte. Electrochem. Commun. **55**, 22–25 (2015). 10.1016/j.elecom.2015.03.010

[52] M. Lécuyer, M. Deschamps, D. Guyomard, J. Gaubicher, P. Poizot, Electrochemical assessment of indigo carmine dye in lithium metal polymer technology. Molecules **26**, 3079 (2021). 10.3390/molecules26113079 34064063 10.3390/molecules26113079 PMC8196690

[53] W. Wei, L. Li, L. Zhang, J. Hong, G. He, An all-solid-state Li-organic battery with quinone-based polymer cathode and composite polymer electrolyte. Electrochem. Commun. **90**, 21–25 (2018). 10.1016/j.elecom.2018.03.006

[54] S. Muench, R. Burges, A. Lex-Balducci, J.C. Brendel, M. Jäger et al., Printable ionic liquid-based gel polymer electrolytes for solid state all-organic batteries. Energy Storage Mater. **25**, 750–755 (2020). 10.1016/j.ensm.2019.09.011

[55] J. Zhang, Z. Chen, Q. Ai, T. Terlier, F. Hao et al., Microstructure engineering of solid-state composite cathode via solvent-assisted processing. Joule **5**, 1845–1859 (2021). 10.1016/j.joule.2021.05.017

[56] F. Hao, X. Chi, Y. Liang, Y. Zhang, R. Xu et al., Taming active material-solid electrolyte interfaces with organic cathode for all-solid-state batteries. Joule **3**, 1349–1359 (2019). 10.1016/j.joule.2019.03.017

[57] X. Zhou, Y. Zhang, M. Shen, Z. Fang, T. Kong et al., A highly stable Li-organic all-solid-state battery based on sulfide electrolytes. Adv. Energy Mater. **12**, 2103932 (2022). 10.1002/aenm.202103932

[58] Z. Yang, F. Wang, Z. Hu, J. Chu, H. Zhan et al., Room-temperature all-solid-state lithium–organic batteries based on sulfide electrolytes and organodisulfide cathodes. Adv. Energy Mater. **11**, 2102962 (2021). 10.1002/aenm.202102962

