# Supplementary material for: A Solvent-Free Covalent Organic Framework Single-Ion Conductor Based on Ion–Dipole Interaction for All-Solid-State Lithium Organic Batteries
Source: Nanomicro Lett. 2024 Aug 9;16:265. doi: 10.1007/s40820-024-01485-3 (PMC11315829; doi:10.1007/s40820-024-01485-3)
Supplement: Supplementary file 1 — Supplementary file1 (DOCX 3519 KB) [file 40820_2024_1485_MOESM1_ESM.docx]

Supporting Information

**A Solvent-Free Covalent Organic Framework Single-Ion Conductor Based on Ion-Dipole Interaction for All-Solid-State Lithium Organic Batteries**

Zhongping Li,^1,2^ Kyeong-Seok Oh,^1^ Jeong-Min Seo,^2^ Wenliang Qin,^3^ Soohyoung Lee,^4^ Lipeng Zhai, ^3,^* Changqing Li,^2^ Jong-Beom Baek,^2,^* and Sang-Young Lee^1,^*

^1^ Department of Chemical and Biomolecular Engineering, Yonsei University, 50 Yonsei-ro, Seodaemun-gu, Seoul, 03722, Republic of Korea.

^2^ School of Energy and Chemical Engineering, Ulsan National Institute of Science and Technology (UNIST), 50 UNIST-gil, Eonyang-eup, Ulju-gun, Ulsan, 44919, Republic of Korea.

^3^ Henan Key Laboratory of Functional Salt Materials, Center for Advanced Materials Research, Zhongyuan University of Technology, Zhengzhou 450007, P. R. China.

^4^ Department of Battery Conflation Engineering, Yonsei University, 50, Yonsei-ro, Seodaemun-gu, Seoul 03772, Republic of Korea.

*Corresponding author. E-mail: [zhailp@zut.edu.cn](mailto:zhailp@zut.edu.cn); jbbaek@unist.ac.kr; syleek@yonsei.ac.kr

**S1 Characterization**

Fourier transform infrared (FTIR) spectra were collected using a Bruker ALPHA Laser class 1, and the powder X-ray diffraction (PXRD) patterns were recorded on a Rigaku (SmartLab) operated at 40 kV and 200 mA with Cu Kα radiation (*λ* = 1.54056 Å) in the range of 2*θ* = 1.5–30 deg. Solid-state nuclear magnetic resonance (NMR) experiments were conducted using an Agilent VNMRS 600 MHz NMR spectrometer at room temperature. Nitrogen sorption isotherms were measured at 77 K using BELSORP. The specific surface areas were calculated using the Brunauer-Emmett-Teller (BET) method. Energy dispersive spectrometry (EDS) mapping images were obtained using a field emission scanning electron microscope (JEOL-7800F), and the surface and cross-sectional morphologies of the electrodes were examined using field emission secondary electron microscopy (FE-SEM, S-4800, Hitachi).

**S2 Density functional theory (DFT) calculations**

For the DFT calculations, the unit cell structure of COF-SO_3_Li was constructed^S1^. The hydrogen atoms of sulfonic acids were substituted with Li atoms and geometry optimization was performed to determine their stable positions using a 1 × 1 × 2 supercell model. All DFT calculations were performed using the DMol3 program^S2–S4^. The generalized gradient approximation with Perdew-Burke-Ernzerhof functional (GGA-PBE) was used for the exchange-correlation energy^S5^. To account for the van der Waals interactions, the semi-empirical Tkatchenko-Scheffler (TS) scheme was included for dispersion correction^S6^, and the DFT semi-core pseudopotential was used for the core-electron treatment. The Brillouin zone was sampled using the Monkhorst-Pack grid as the Γ-point for all systems^S7^. The SCF convergence for each electronic energy was set to 1.0 × 10^–5^ Ha, and the geometry optimization convergence criteria were set to as follows: 1.0 × 10^–5^ Ha for energy, 0.002 Ha Å^–1^ for force, and 0.005 Å for displacement. Further, Li^+^ migration paths were studied using linear and quadratic synchronous transit (LST/QST) methods in combination with the conjugated gradient (CG) refinement^S8^. Lastly, the adsorption energies (*E*_ads_) were calculated using *E*_ads_= *E*_ad/sub_ -*E*_ad_ -*E*_sub_, where *E*_ad_/_sub_, *E*_ad_, and *E*_sub_ are the total energies of the optimized adsorbate/substrate system, the adsorbate in the structure, and the clean substrate, respectively.

**S3 Fabrication of ASSLOBs with Li-COF-2@P75%.**

Versatile COF-embedded cathode mixtures were prepared with a composition of 5,5′-dimethyl-2,2′-bis-p-benzoquinone (Me_2_BBQ)/carbon black (CB)/polyvinylidene fluoride (PVDF)/Li-COF-2@P_75%_ = 26.5/51/8.5/15 (w/w/w/w) in *N*-methyl-2-pyrrolidone (NMP) with an areal-mass-loading level = ~1.0 mg cm^−2^. Thereafter, the slurry mixturein NMP was cast on an Al foil, and the COF-embedded cathode slurry was vacuum-dried at 120 °C for 12 h, followed by pressing at 5 MPa at 120 °C for 1 h. Thereafter, the cathode was solidified after exposure to UV irradiation (Hg UV-lamp, Lichtzen) for less than 1 min to crosslink the PEGDA polymer backbone^S9−S11^ at an irradiation peak intensity of approximately 3000 mW cm^−2^.

To fabricate the control cathode, a slurry mixture containing the Me_2_BBQ/CB/PVDF in NMP was cast on an Al foil. The control cathode slurry was vacuum-dried at 120 °C for 12 h, followed by roll-pressing. For this comparison, the control cathodes were prepared with the same ratio of each component (except electrolytes) as the COF-embedded electrode and an areal-mass-loading of ~1 mg cm^−2^.

A Li-metal foil (Honjo Metal Co., Ltd., 100 µm) was used as the anode. The electrochemical performance of the COF-embedded cathode was characterized using a 2032 coin-type cell (= COF-embedded cathode (containing a Li-COF-2@P_75%_)|Li-COFs@P_75%_|Li), whereas the control cathode was prepared using the same type of cell (= control cathode (containing a liquid electrolyte)|polyethylene (PE) separator (16 µm)|Li, electrolyte: 1 M LiTFSI in DOL/DME = 1/1 (v/v) (for Me_2_BBQ||Li). All the cells were assembled in an argon-filled glove box.

**S4 Electrochemical characterization**

The ionic conductivity of the electrolytes was estimated by conducting electrochemical impedance spectroscopy (EIS) analysis in the frequency range of 10^−2^ to 10^6^ Hz and an applied amplitude of 10 mV using a potentiostat (VSP classic, Bio-Logic). The Li^+^ transference number (*t*_Li⁺_) was evaluated using a potentiostatic polarization method. The DC polarization through a Li^+^ non-blocking symmetric cell and its sequential EIS before and after the polarization were analyzed to determine the Li^+^ transference number^S9−S11^:

$$t_{Li+}=\frac{I_{s}(\Delta V-I_{o}R_{o})}{I_{o}(\Delta V-I_{s}R_{s})}$$

where ΔV is the applied potential, *I*_o_ and *R*_o_ are the initial current and resistance, respectively, and *I*_s_ and *R*_s_ are the steady-state current and resistance after the polarization, respectively. Linear sweep voltammetry (LSV) was conducted using an asymmetric cell (SUS||Li) at a sweep rate of 0.1 mV s^−1^. The Li metal anode cycling test was performed using the Li||Li symmetric cells at a current density of 0.05 mA cm^−2^ and an area capacity of 0.25 mAh cm^−2^ at 25 °C. The electrochemical performance of the coin-type cells (Me_2_BBQ cathodes |Li-COFs@P_75%_|Li metal (100 µm) anode) was examined using a cycle tester (PEBC050.1, PNE Solution Co.) in the voltage range of 1.7–3.4 vs. Li/Li^+^.

**Supplementary Figures and Tables**


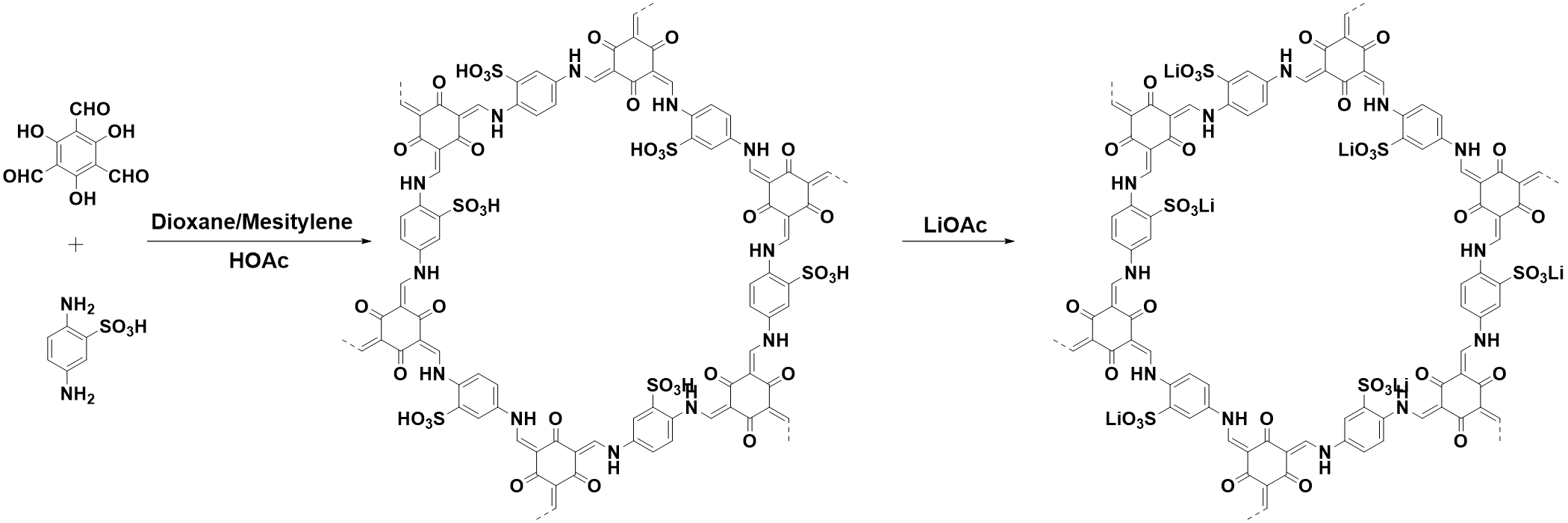


**Fig. S1.** Synthesis of Li-COF-1.


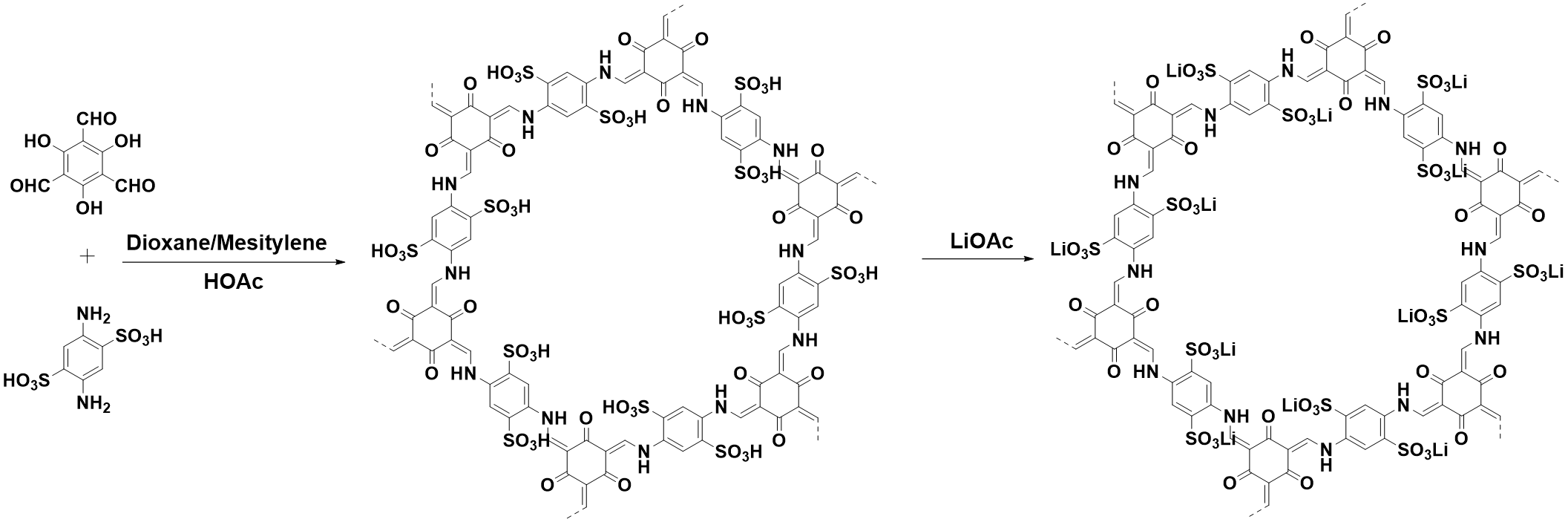


**Fig. S2.** Synthesis of Li-COF-2.


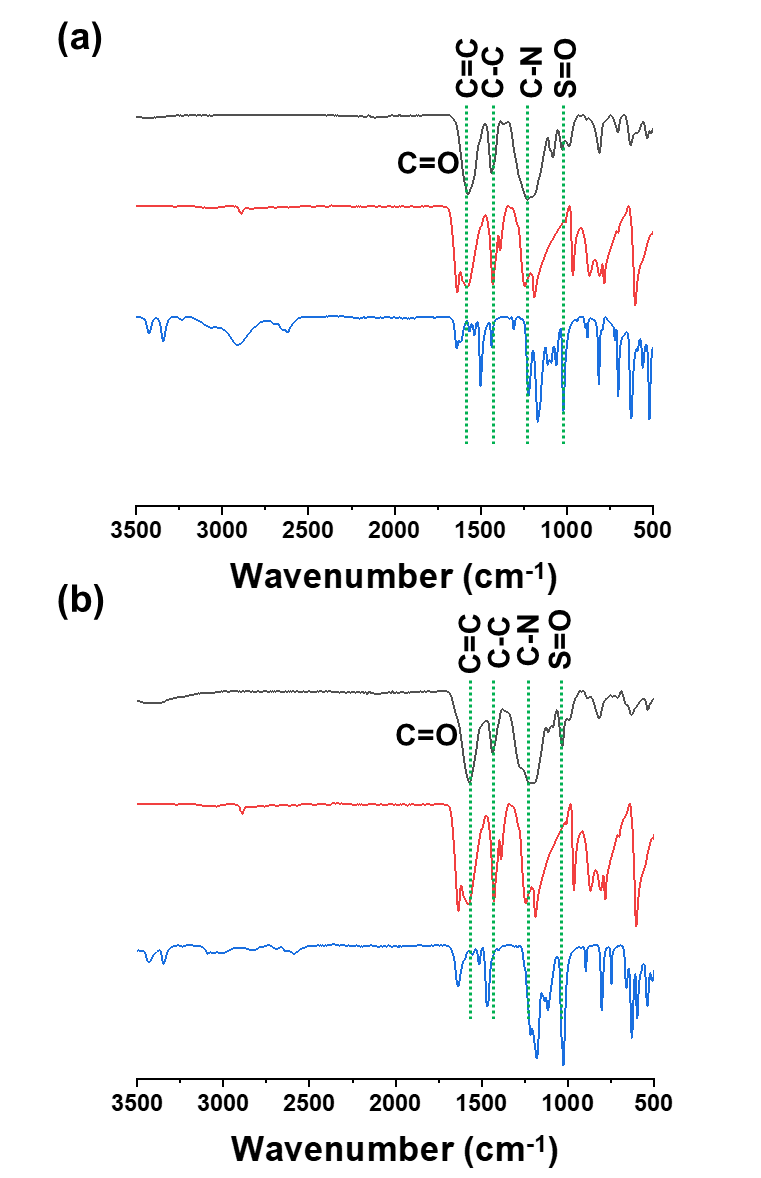


**Fig. S3.** FT-IR spectra of (a) Li-COF-1 and (b) Li-COF-2 (COFs: black; 2,4,6-triformylphloroglucinol: red; 2,5-diaminobenzenesulfonic acid or 2,5-diaminobenzene-1,4-disulfonic acid: blue).

The stretching vibration signals of the keto unit (C=O), carbon–carbon double bond (C=C), carbon–nitrogen single bond (C–N), and oxygen sulfur bond (O=S) were observed in the FT-IR analyses of the Li-COF-1 (Fig. S3a) and Li-COF-2 (Fig. S3b), respectively, indicating the presence of β-ketoenamine linkage.


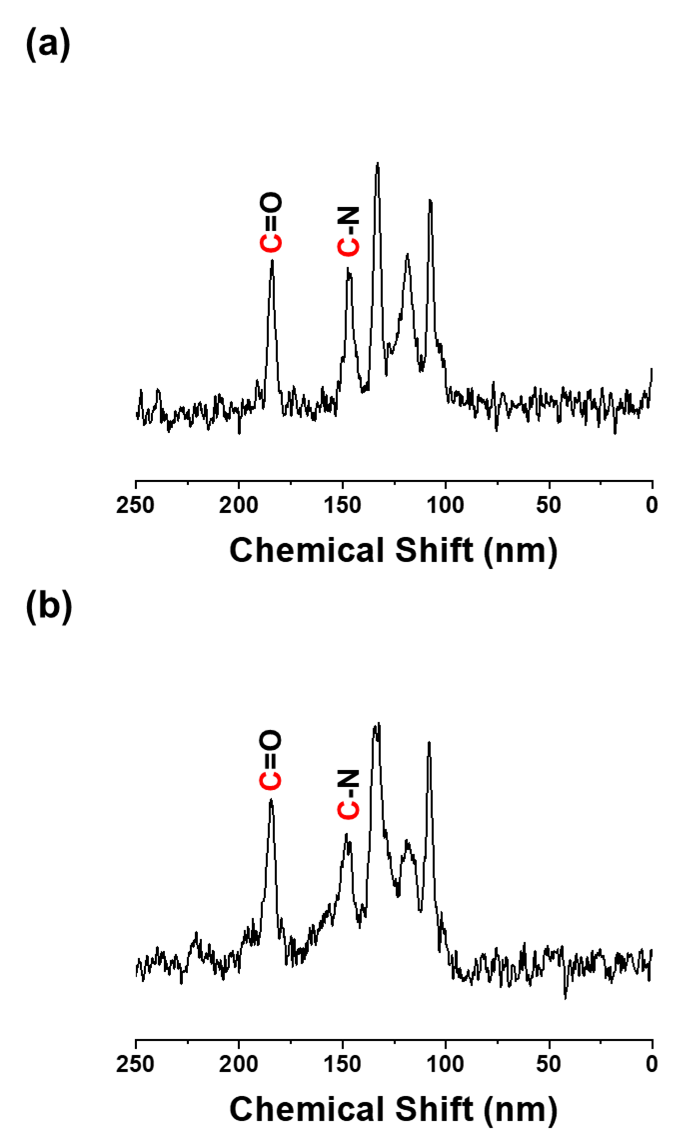


**Fig. S4.** ^13^CNMR spectra of (a) Li-COF-1 and (b) Li-COF-2.

The carbon signals of the keto unit (C=O) were observed at 184 and 184.5 ppm in the NMR spectra of Li-COF-1 (Fig. S4a) and Li-COF-2 (Fig. S4b), respectively.


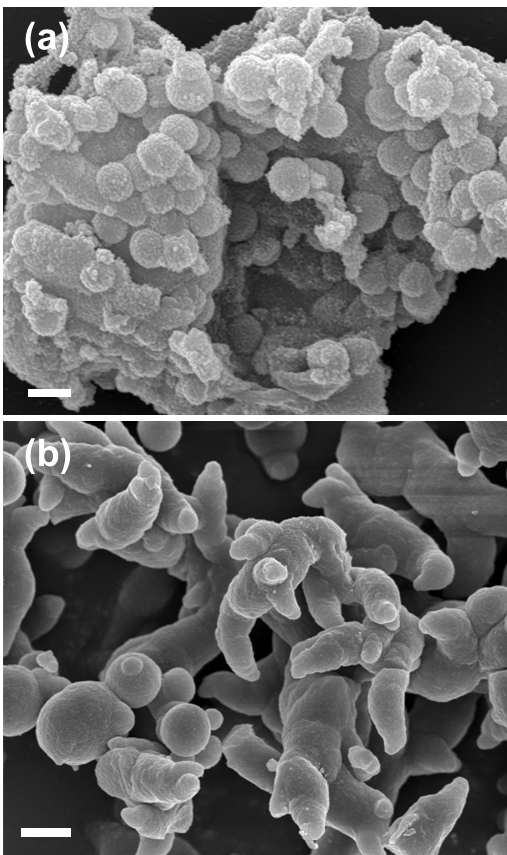


**Fig. S5.** FE-SEM images of (a) Li-COF-1 and (b) Li-COF-2 (scale bar: 1 μm).

FE-SEM (Fig. S5) and EDS mapping analyses (Fig. S6 and S7) suggested the uniform morphology and distribution of carbon, oxygen, nitrogen, and sulfur elements in the Li-COF-1 and Li-COF-2.


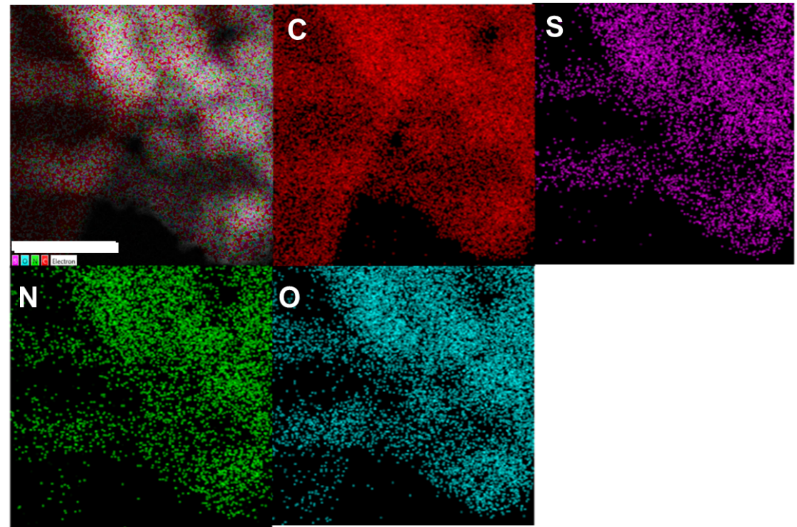


**Fig. S6.** EDS mapping images of Li-COF-1 (scale bar: 1 μm).


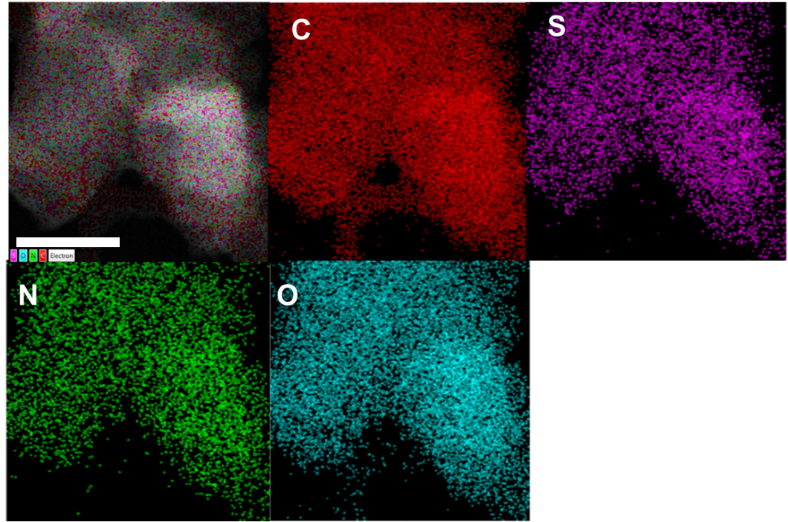


**Fig. S7.** EDS mapping images of Li-COF-2 (scale bar: 1 μm).

**
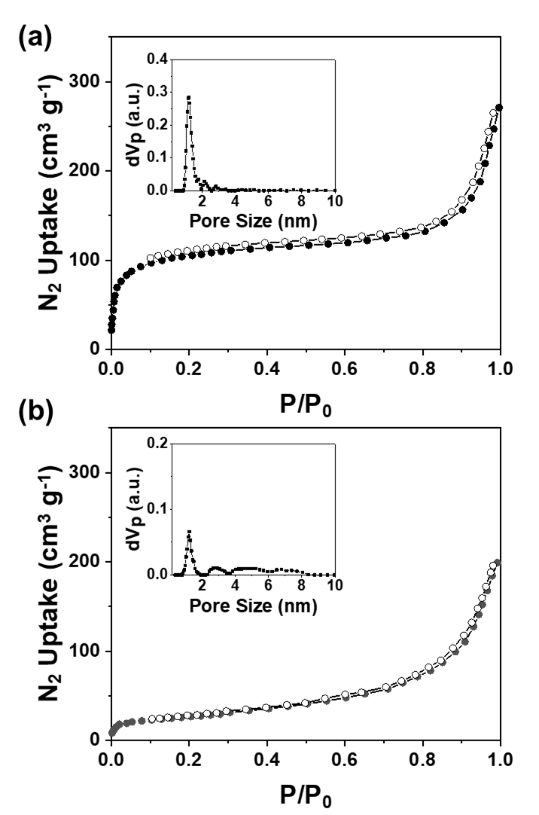
**

**Fig. S8.** Nitrogen adsorption–desorption isotherms of (a) Li-COF-1 and (b) Li-COF-2 measured at 77 K (inset: pore size distribution of Li-COFs).

**
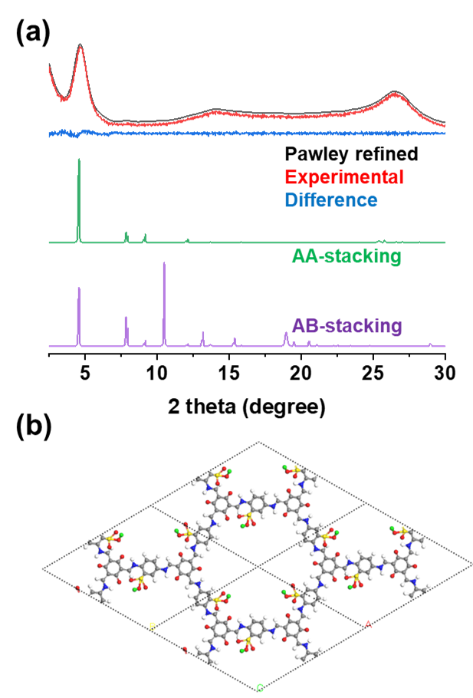
**

**Fig. S9.** (a) PXRD patterns of Li-COF-1. (b) Unit cell of Li-COF-1 (a = b = 22.6542 Å, b = 21.9491, c = 3.4794 Å; *R*_p_ = 1.84, *R*_wp_ = 1.65).

**
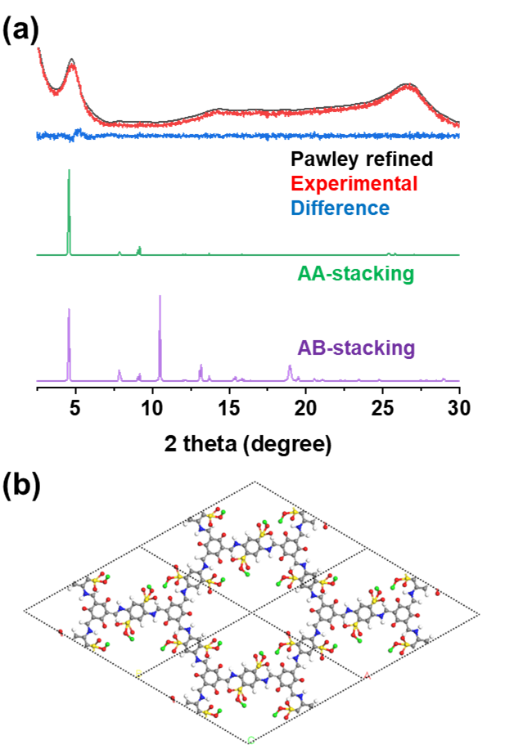
**

**Fig. S10.** (a) PXRD patterns of Li-COF-2. (b) Unit cell of Li-COF-2. (a = 22.6542, b = 22.1314 Å, c = 3.5313 Å; *R*_p_ = 2.77, *R*_wp_ = 2.08).


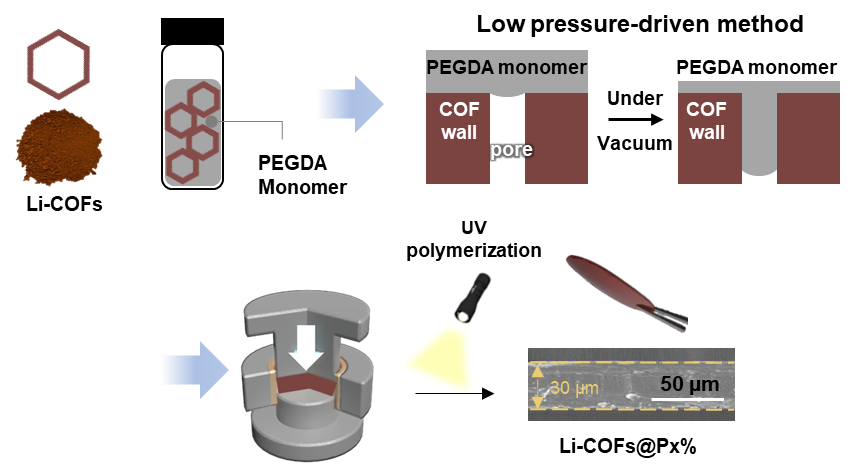


**Fig. S11.** Schematic illustration depicting the fabrication process of Li-COFs@P_X_% using the low pressure-driven method.


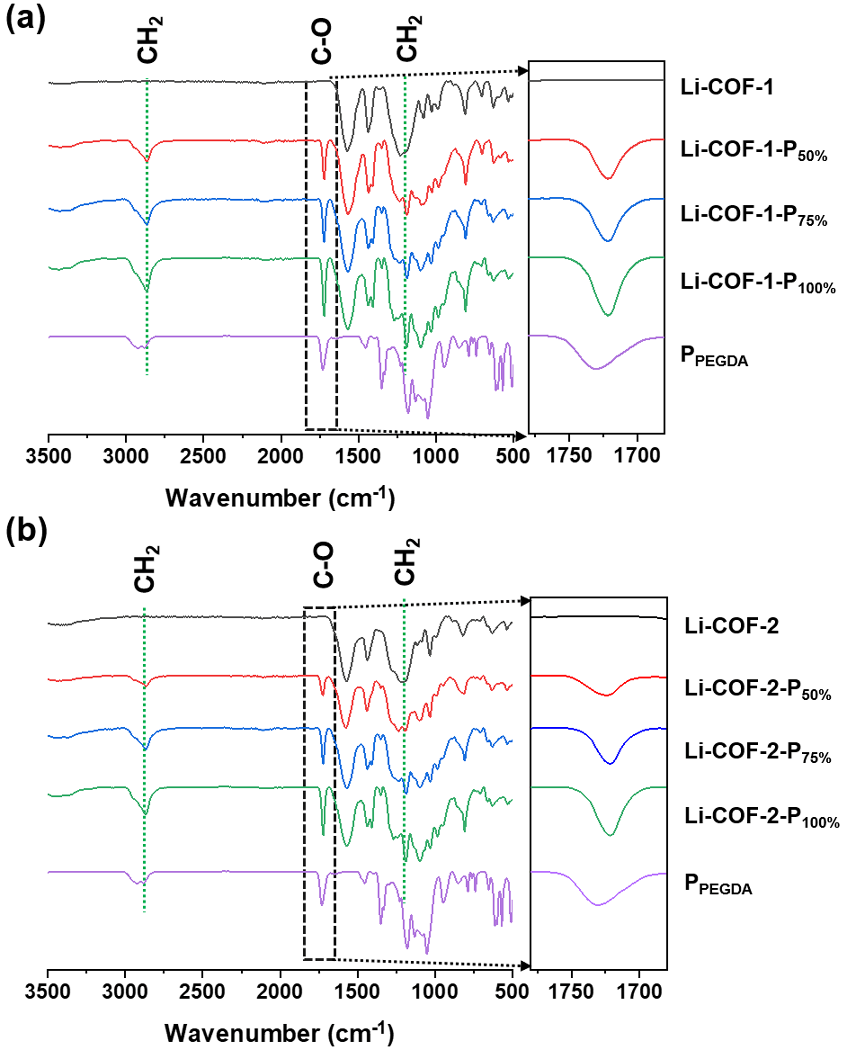


**Fig. S12.** FT-IR spectra of Li-COFs, Li-COF-1@Px_%_, Li-COF-2@Px_%_, and P_PEGDA_ powders.

**
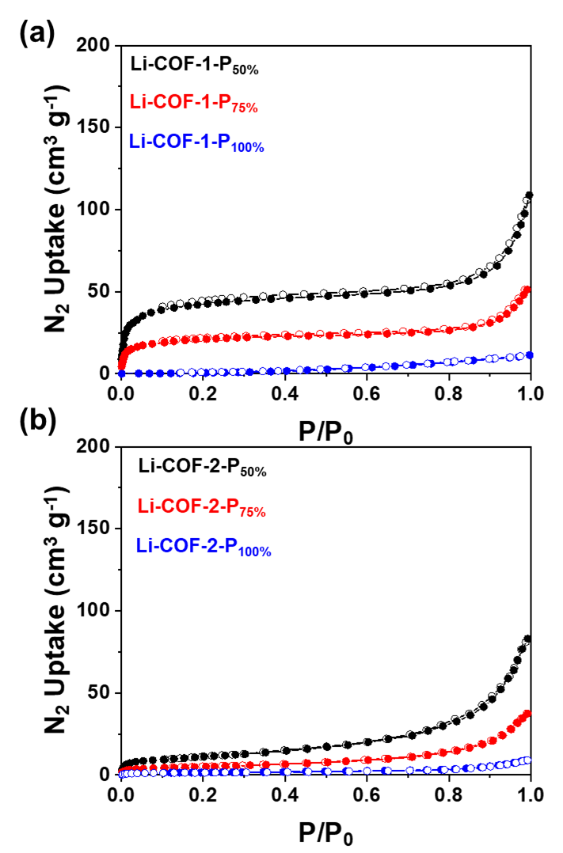
**

**Fig. S13.** Nitrogen adsorption–desorption isotherms of (a) Li-COF-1@Px_%_ and (b) Li-COF-1@Px_%_ powders measured at 77 K.

**
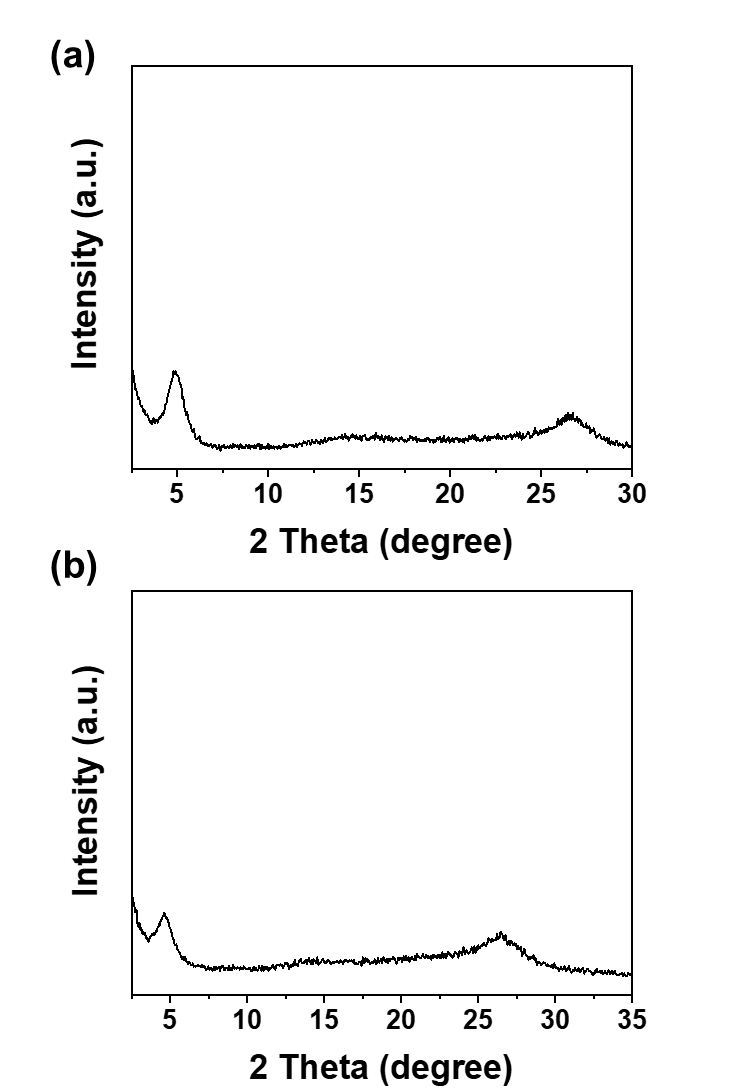
**

**Fig. S14.** PXRD patterns of (a) Li-COF-1@P_100%_ and (a) Li-COF-2@P_100%_ powders.

**
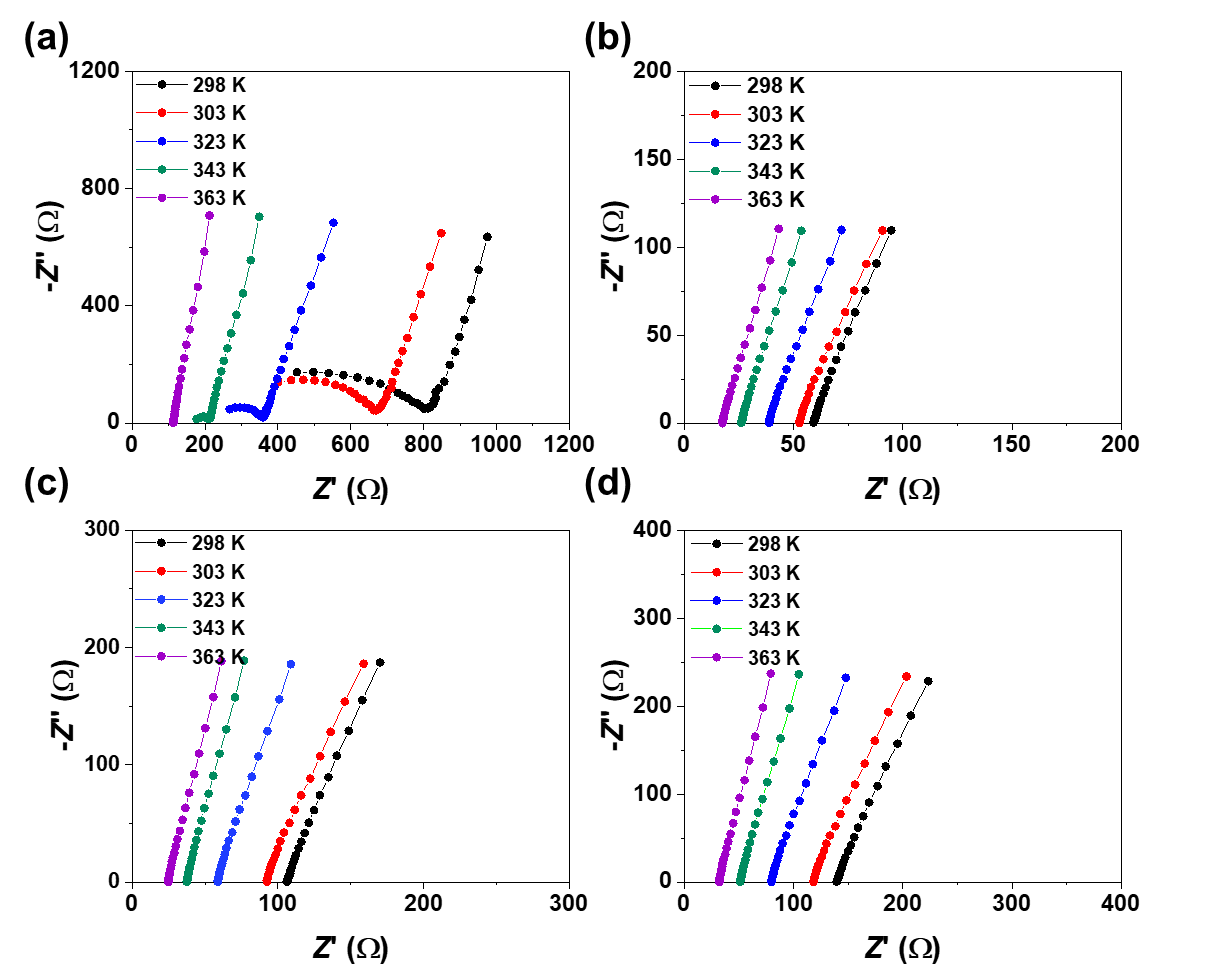
**

**Fig. S15.** Electrochemical impedance spectroscopy (EIS) profiles of (a) Li-COF-1@P_25%_, (b) Li-COF-1@P_50%_, (c) Li-COF-1@P_100%_, and (d) Li-COF-1@P_125%_.


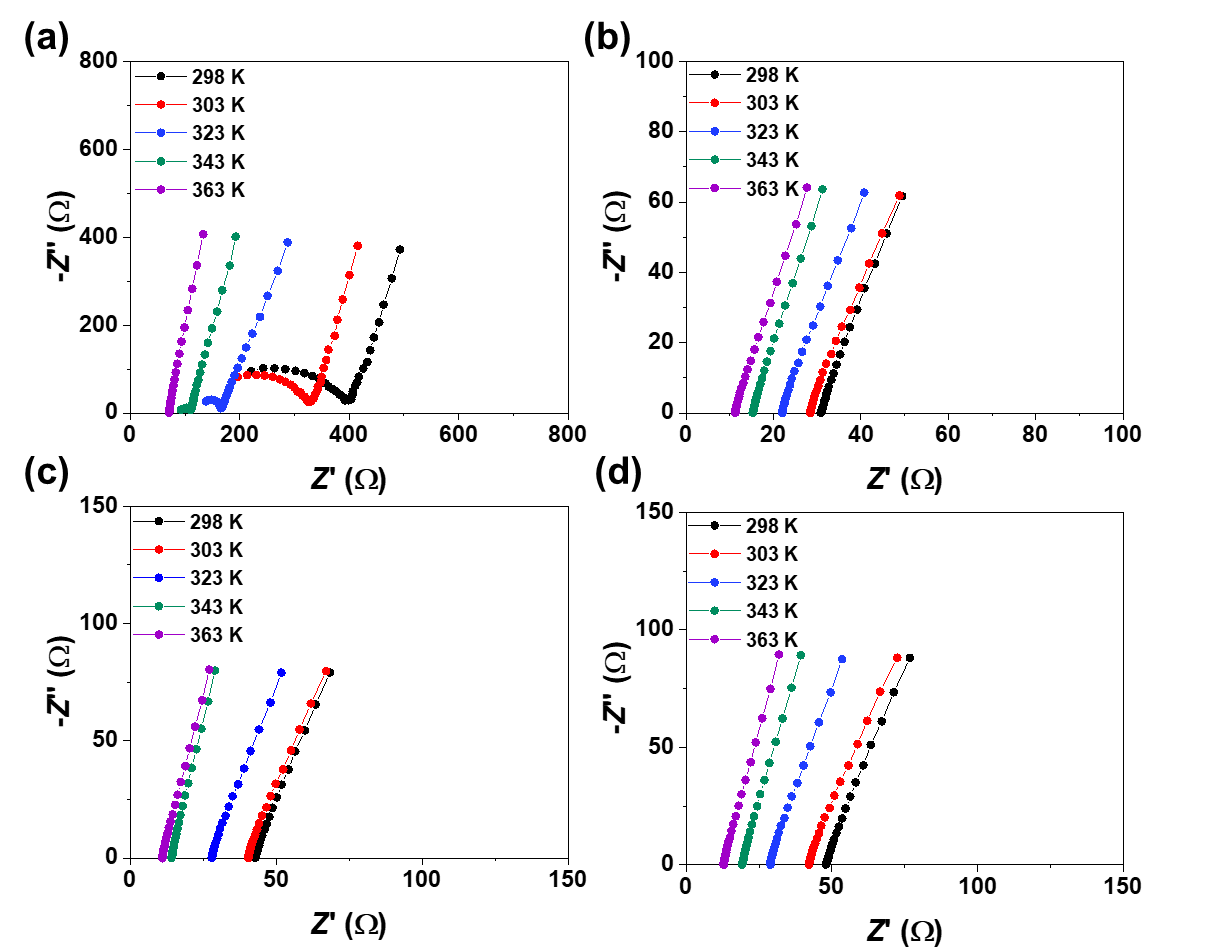


**Fig. S16.** Electrochemical impedance spectroscopy (EIS) profiles of (a) Li-COF-2@P_25%_, (b) Li-COF-2@P_50%_, (c) Li-COF-2@P_100%_, and (d) Li-COF-2@P_125%_.


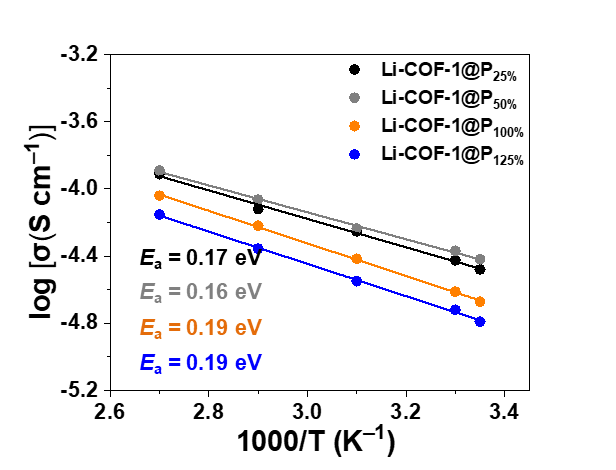


**Fig. S17.** Arrhenius plots of Li-COF-1@P_25%_, Li-COF-1@P_50%_, Li-COF-1@P_100%_, and Li-COF-1@P_125%_.


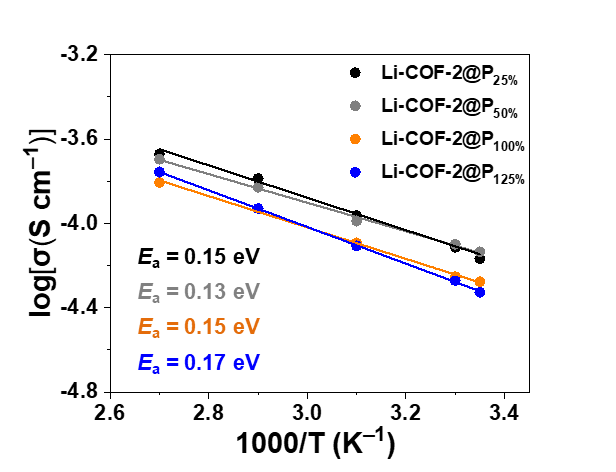


**Fig. S18.** Arrhenius plots of Li-COF-2@P_25%_, (c) Li-COF-2@P_50%_, Li-COF-2@P_100%_, and Li-COF-2@P_125%_.

**
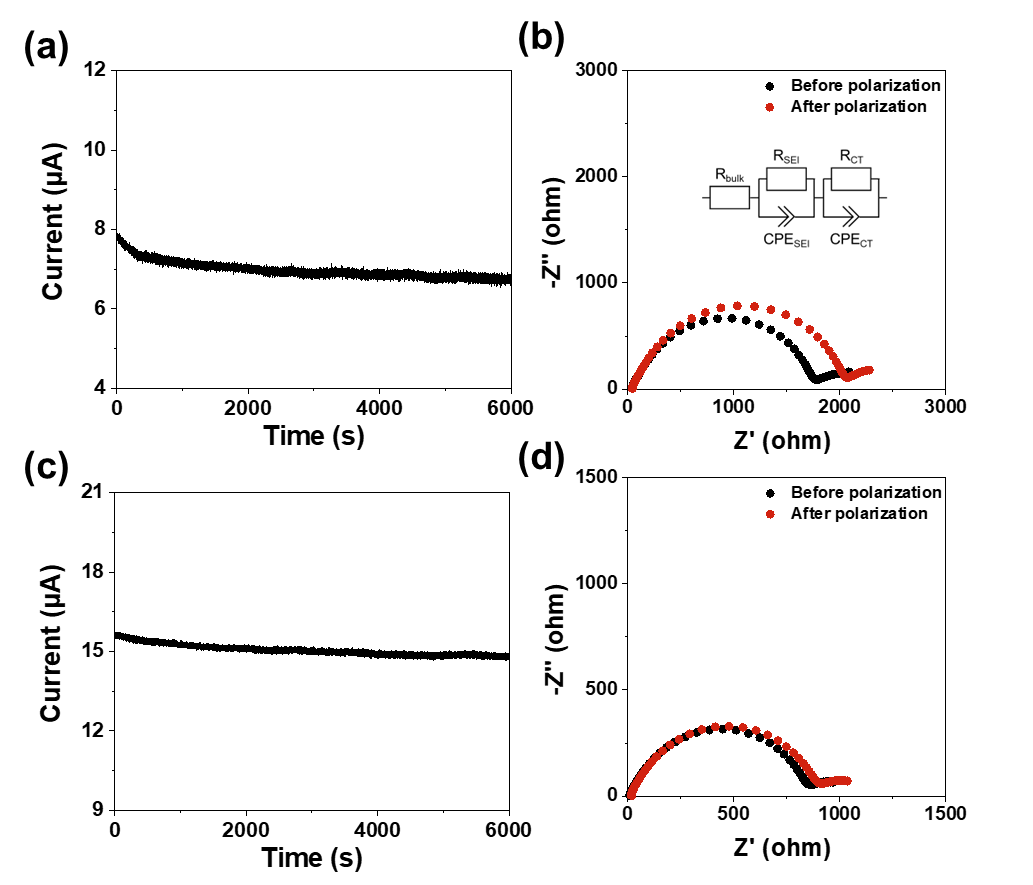
**

**Fig. S19.** Time-dependent current profile of the Li||Li symmetric cell containing (a) Li-COF-1 and (c) Li-COF-1@P_75%_ at 10 mV polarization. EIS profiles of (b) Li-COF-1 and (d) Li-COF-1@P_75%_ (the inset shows corresponding equivalent circuit model)_._

**
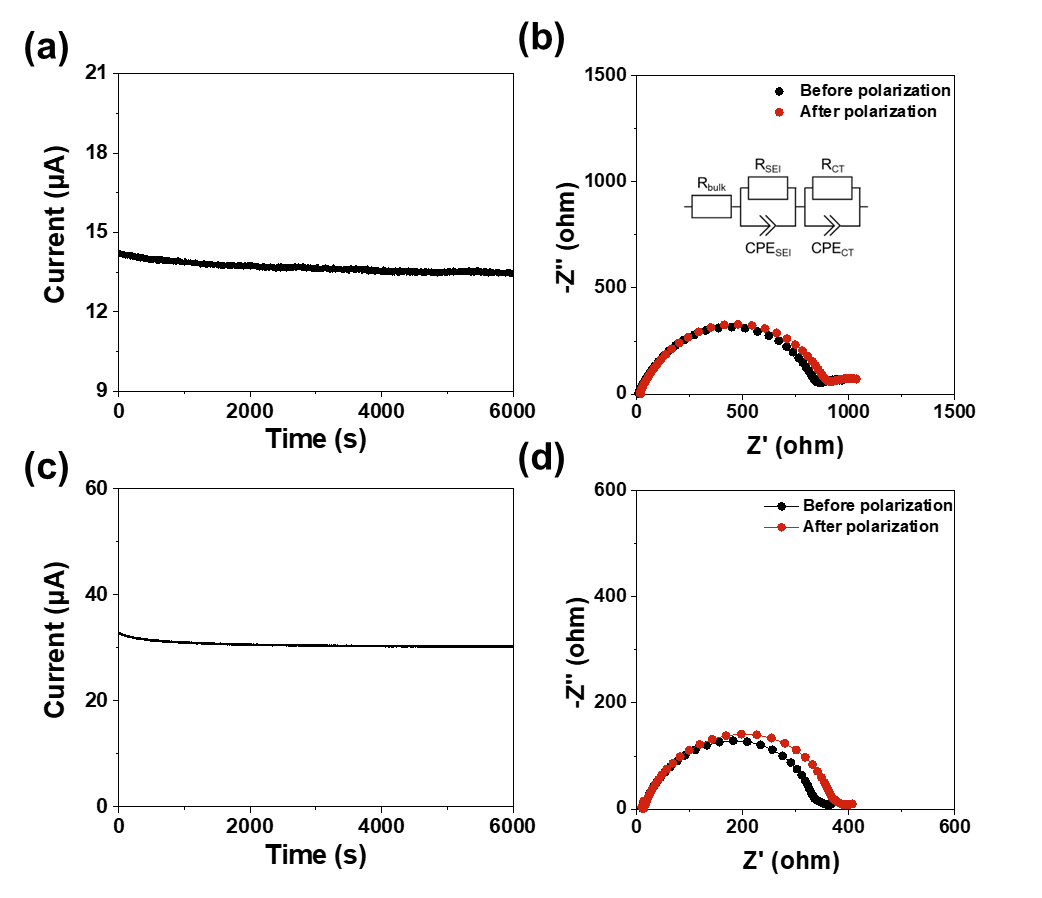
**

**Fig. S20.** Time-dependent current profile of the Li||Li symmetric cell containing (a) Li-COF-2 and (c) Li-COF-2@P_75%_ at 10 mV polarization. EIS profiles of (b) Li-COF-2 and (d) Li-COF-2@P_75%_ (the inset shows corresponding equivalent circuit model)_._

**
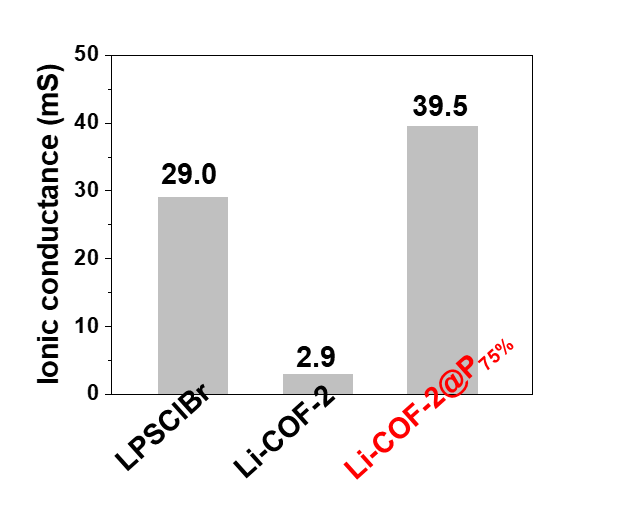
**

**Fig. S21.** Comparison of the ionic conductance of Li-COF-2@P_75%_ to those of Li-COF-2 and previously reported 700 μm-thick inorganic Li_6_PS_5_Cl_0.5_Br_0.5_ pellets.

**
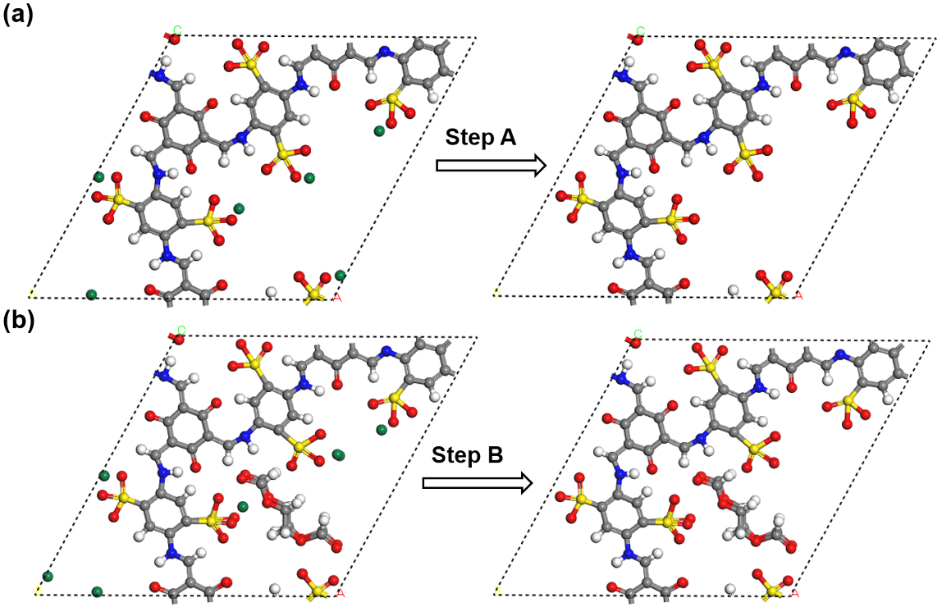
**

**Fig. S22.** Li^+^ dissociation process of (a) Li-COF-2 and (b) Li-COF-2@P_X%_.


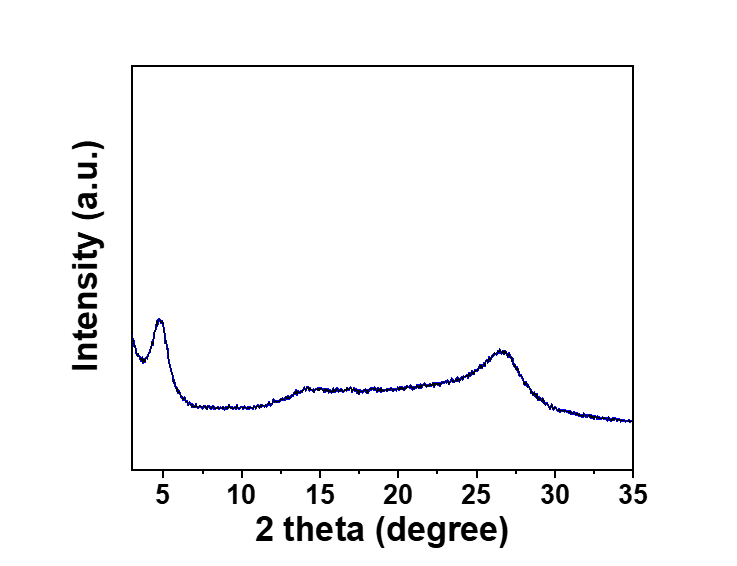


**Fig. S23.** PXRD pattern of Li-COF-2@P_75%_ after the Li||Li symmetric cell test.

**
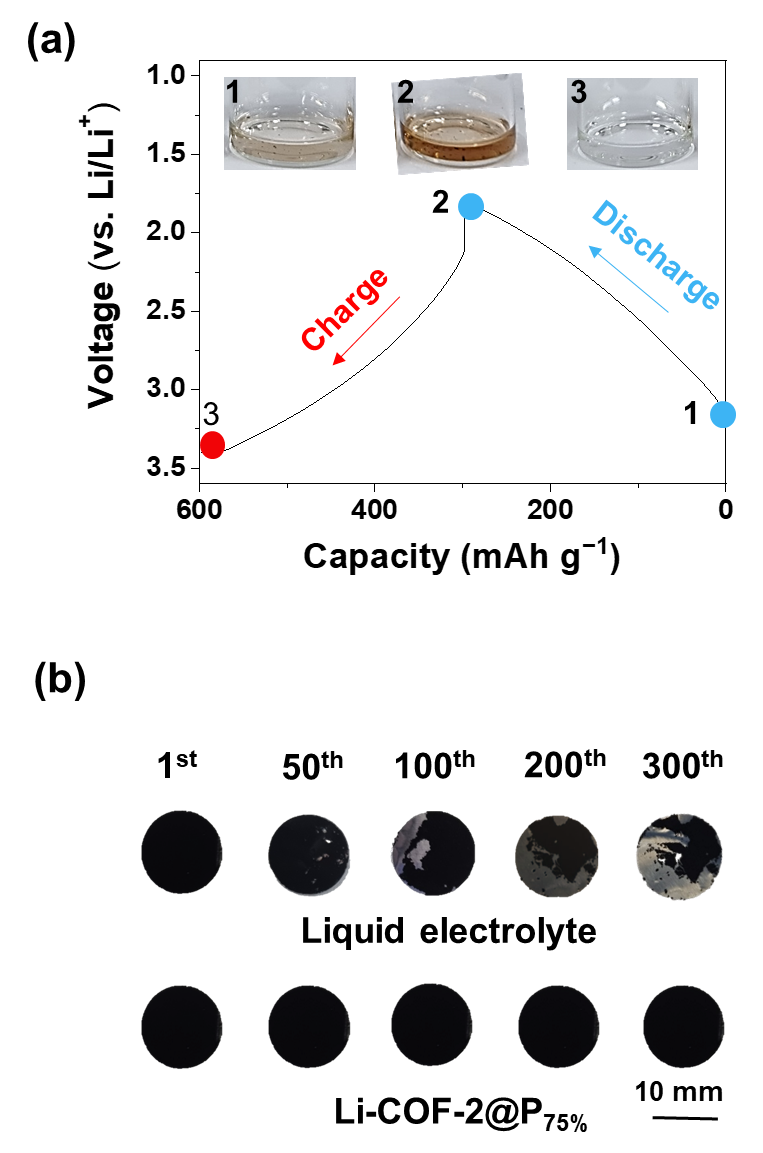
**

**Fig. S24.** Voltage profile of the Me_2_BBQ electrode showing a large solubility difference at different redox states. The electrochemical process was divided into the discharge (1🡪 2) and charge points (2 🡪 3). The color of the electrolyte gradually changed to deep brown as the discharge progressed and became transparent again when it returned to the charged state.

**
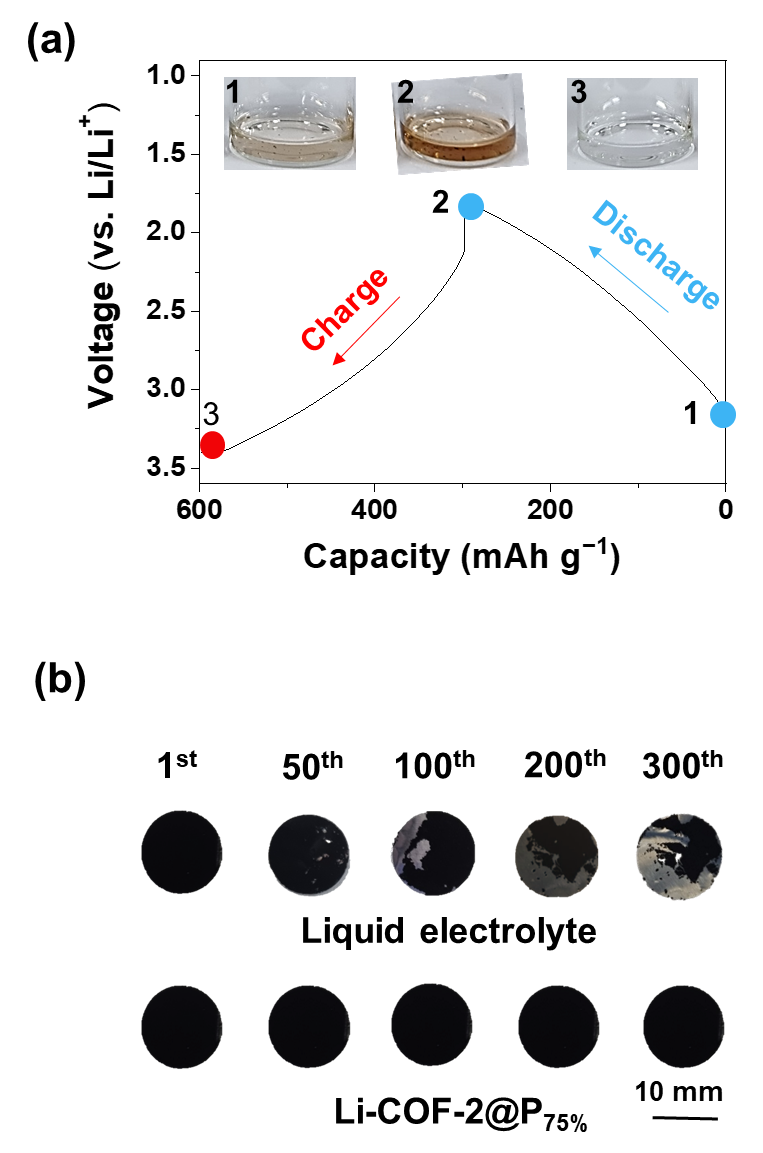
**

**Fig. S25.** Photographs of the Me_2_BBQ cathode as a function of the cycle number.

**
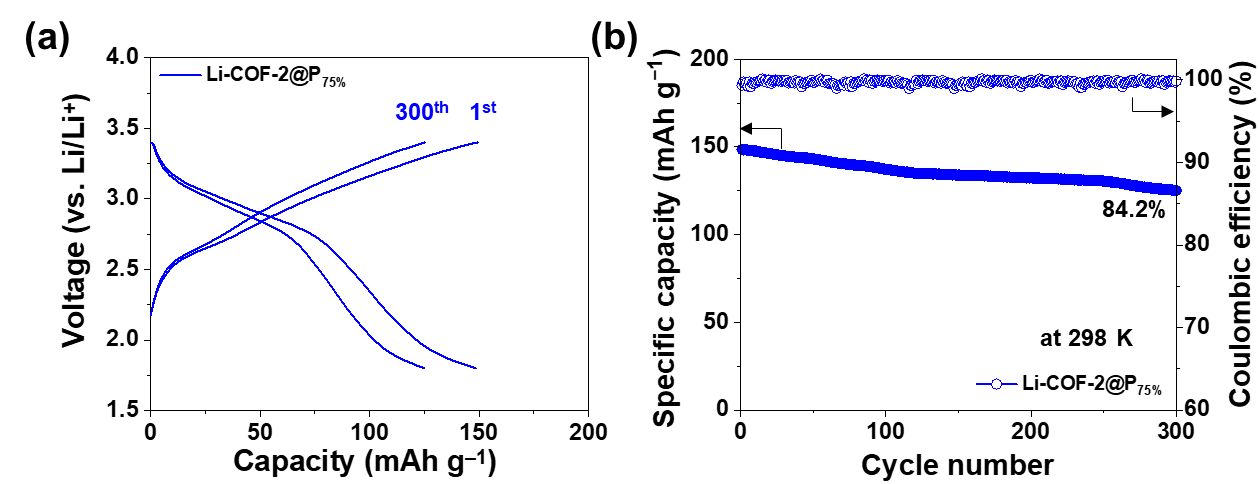
**

**Fig. S26.** (a) Voltage profiles and (b) cycling performance of SSOLBs (Me_2_BBQ‖Li) with Li-COF-2@P_75%_ (vs. liquid electrolyte) at a charge/discharge current density of 5.0/5.0 C and voltage range of 1.8–3.4 V at 25 °C.

**Table S1.** Li amount, ionic conductivity, and *E*_a_ values of the Li-COFs and Li-COFs@Px_%_.

| **Solid EL** | **Name** | **Content of implanted polymer in pore of COF (%)** | **Experiment**  **Li amount**  **(w%)** | **Thin film** | **Ionic conductivity (S cm^-1^, RT)** | ***E*_a_ (eV)** |
| --- | --- | --- | --- | --- | --- | --- |
| **Li-COF-1@P_X%_** | Li-COF-1 | 0 | 2.29 | X | 2.7 $\times$ 10^-5^ | 0.18 |
|  | Li-COF-1@P_25%_ | 25 | 2.14 | X | 3.6 $\times$ 10^-5^ | 0.17 |
|  | Li-COF-1@P_50%_ | 50 | 1.97 | ○ | 4.1 $\times$ 10^-5^ | 0.16 |
|  | Li-COF-1@P_75%_ | 75 | 1.83 | ○ | 5.1 $\times$ 10^-5^ | 0.15 |
|  | Li-COF-1@P_100%_ | 100 | 1.71 | ○ | 2.3 $\times$ 10^-5^ | 0.19 |
|  | Li-COF-1@P_125%_ | 125 | 1.58 | ○ | 1.8 $\times$ 10^-5^ | 0.19 |
| **Li-COF-2@ _X%_** | Li-COF-2 | 0 | 3.57 | X | 4.9 $\times$ 10^-5^ | 0.16 |
|  | Li-COF-2@P_25%_ | 25 | 3.36 | X | 7.2 $\times$ 10^-5^ | 0.15 |
|  | Li-COF-2@P_50%_ | 50 | 3.17 | ○ | 7.8 $\times$ 10^-5^ | 0.13 |
|  | Li-COF-2@P_75%_ | 75 | 3.05 | ○ | 8.9 $\times$ 10^-5^ | 0.11 |
|  | Li-COF-2@P_100%_ | 100 | 2.92 | ○ | 5.7 $\times$ 10^-5^ | 0.15 |
|  | Li-COF-2@P_125%_ | 125 | 2.79 | ○ | 5.1 $\times$ 10^-5^ | 0.17 |

**Table S2**. Comparison in the synthesis conditions and ion conduction characteristics between the Li-COF-2@P_75%_ (this study) and previously reported organic single ion conducting electrolytes.

| **Classification** | **Sample** | **Ionic** **conduction**  **(S cm^-1^)** | ***E*_a_ (eV)** | ***t*_Li+_** | **Ref.** |
| --- | --- | --- | --- | --- | --- |
| COFs | Li-COF-2@P_75%_ | 8.9 $\times$ 10^−5^  (298 K) | 0.11 | 0.95 | ***This work*** |
|  | TpPa-SO_3_Li | 2.7 $\times$ 10^−5^  (298 K) | 0.18 | 0.9 | Ref. 8 |
|  | LiCON-1 | 2.13 × 10^−7^  (295 K) | 0.25 | 0.86 | Ref. 12 |
|  | LiCON-2 | 4.36 × 10^−6^  (295 K) | 0.22 | 0.83 |  |
|  | LiCON-3 | 3.2 $\times$ 10^−5^  (295 K) | 0.13 | 0.92 |  |
|  | LiOOC-COF1 | 7.23 $\times$ 10^−7^  (303 K) | 0.21 | 0.82 | Ref. 13 |
|  | LiOOC-COF2 | 2.66 × 10^−6^  (303 K) | 0.2 | 0.86 |  |
|  | LiOOC-COF3 | 1.3 $\times$ 10^−5^  (303 K) | 0.17 | 0.91 |  |
|  | LiO_3_S-COF2 | 4.46×10^−5^  (295 K) | 0.18 | 0.93 | Ref. 14 |
|  | LiO_3_S-COF2 | 5.47×10^−5^  (303 K) |  |  |  |
| Polymer  Electrolyte | P(STFSILi)  Triblock copolymer | 1.3 $\times$ 10^−5^  (358 K) | - | 0.85 | Ref. 15 |
|  | LiPBPAB  Polymeric borate/PEO blend | 1.7 $\times$ 10^−7^  (298 K) | - | 0.98 | Ref. 16 |
|  | LiBC-1  Comb-branched copolymer | 2.3 $\times$ 10^−6^  (298 K) | - | 0.83 | Ref. 17 |
|  | PEO*_35K_* macro-CTA  Triblock copolymer | 1.0 $\times$ 10^−5^  (298 K) | - | 0.91 | Ref. 18 |
|  | PEO-LiPCSI | 7.3 $\times$ 10^−5^ | - | 0.84 | Ref. 19 |

**Table S3**. Comparison of the *E_a_* values of previously reported COF-based electrolytes.

| **Classification** | **Sample** | ***E*_a_ (eV)** | **Ref.** |
| --- | --- | --- | --- |
| **Acting as solid-state single ion conducting electrolytes** | Li-COF-2@P_75%_ | 0.11 | ***This work*** |
|  | TpPa-SO_3_Li | 0.18 | Ref. 8 |
|  | LiCON-1 | 0.25 | Ref. 12 |
|  | LiCON-2 | 0.22 |  |
|  | LiCON-3 | 0.13 |  |
|  | LiOOC-COF1 | 0.21 | Ref. 13 |
|  | LiOOC-COF2 | 0.2 |  |
|  | LiOOC-COF3 | 0.17 |  |
|  | LiO_3_S-COF2 | 0.18 | Ref. 14 |
| **Acting as single ion conducting electrolyte containing solvents** | CD-COFs | 0.26 | Ref. 20 |
|  | p-PaSO_3_Li-COF | 0.13 | Ref. 21 |
|  | CH_3_-Li-ImCOF | 0.27 | Ref. 22 |
|  | H-Li-ImCOF | 0.12 |  |
|  | CF_3_-Li-ImCOF | 0.10 |  |
| **Acting as solid electrolytes containing**  **additional Li salts** | COF-5 | 0.34 ± 0.04 | Ref. 23 |
|  | Li^+^@TPB-TP-COF | 1.05 | Ref. 24 |
|  | Li^+^@TPB-BMTP-COF | 0.87 |  |
|  | Li-CON-TFSI | 0.34 | Ref. 25 |
|  | COF-PEO-3-Li | 1.07 | Ref. 26 |
|  | COF-PEO-6-Li | 0.92 |  |
|  | COF-PEO-9-Li | 0.73 |  |
|  | Im-COF-TFSI@Li | 0.32 | Ref. 27 |
|  | Li^+^@TPB-DMTP-COF | 0.96 | Ref. 28 |
|  | Li^+^@[TEO]_0.33_-TPB-DMTP-COF | 0.78 |  |
|  | Li^+^@[TEO]_0.5_-TPB-DMTP-COF | 0.68 |  |
|  | Li^+^@TPB-BMTP-COF | 0.87 |  |
|  | Li^+^@[TEO]_0.33_-TPB-BMTP-COF | 0.82 |  |
|  | TPB-DHTP-COF@Li | 1.08 | Ref. 29 |
|  | dCOF-NH_2_-60@Li | 0.81 |  |
|  | dCOF-ImBr-60 | 0.67 |  |
|  | dCOF-ImTFSI-20 | 0.49 |  |
|  | dCOF-ImTFSI-40@Li | 0.41 |  |
|  | dCOF-ImTFSI-60@Li | 0.28 |  |
|  | COF-PEG-B1-Li | 0.74 | Ref. 30 |
|  | COF-PEG-B3-Li | 0.71 |  |
|  | COF-PEG-B6-Li | 0.60 |  |
|  | PEG-Li@NKCOF-11 | 0.18 | Ref. 31 |
|  | COF-MCMC | 0.31 | Ref. 32 |
| **Acting as electrolytes containing**  **additional Li salts and solvents** | PEG-Li^+^@CD-COF-Li | 0.17 | Ref. 33 |
|  | PEG-Li^+^@COF-300 | 0.20 |  |
|  | PEG-Li^+^@COF-5 | 0.35 |  |
|  | PEG-Li^+^@EB-COF-ClO_4_ | 0.21 |  |
|  | IL-1.0@NUST-7 | 0.317 | Ref. 34 |
|  | IL-1.0@NUST-8 | 0.301 |  |
|  | IL-1.0@NUST-9 | 0.323 |  |

**Table S4**. Fitted values of *R*_Int_ (ohm) obtained from the EIS profiles of the Li||Li symmetric cell with Li-COF-2@P_75%_ (vs. Li-COF-1, Li-COF-1@P_75%_, and Li-COF-2) as a function of cycle time.

| ***R*_Int_** | **0 h** | **50 h** | **100 h** | **250 h** | **500 h** |
| --- | --- | --- | --- | --- | --- |
| **Li-COF-1** | 1713.2 | 1824.9 | 2008.3 | - | - |
| **Li-COF-1@P_75%_** | 604.7 | 611.5 | 618.8 | 632.1 | 639.6 |
| **Li-COF-2** | 842.8 | 867.1 | 909.5 | - | - |
| **Li-COF-2@P_75%_** | 334.5 | 342.3 | 344.7 | 348.4 | 352.3 |

**Table S5** | Comparison in the cell components, operating conditions, and electrochemical performance between the ASSLOBs (this study) and previously reported ASSLOBs.

| **Classification** | **Solid electrolyte** | **Anode** | **Operating**  **temp.** | **Specific capacity**  **(mAh g**^−1^**)** | **Cycling stability**  **(Capacity retention after**  **cycle number at current rate)** | **Capacity fading rate**  **(%/cycle)** | **Ref.** |
| --- | --- | --- | --- | --- | --- | --- | --- |
| **COF** | Li-COF-2@P_75%_ | Li | RT | 299 | 88.3%, 2000 cycles,  20 mA g^−1^ | 0.006 | ***This work*** |
|  | Lithiated COFs with  sulfonate terminal group | Li | 20 ℃ | 200 | 87.7%, 500 cycles,  500 mA g^−1^ | 0.025 | Ref. 12 |
| **Polymer SE** | PMA/PEG-LiClO_4_-SiO_2_ composite | Li | RT | 418 | 95%, 50 cycles, 0.2 C  (89.2 mA g^−1^) | 0.106 | Ref. 35 |
|  | PPC-KFSI + Cellulose  nonwoven backbone | Ka | RT | 118 | 84%, 40 cycles,  10 mA g^−1^ | 0.4 | Ref. 36 |
|  | x-PS@PSTFSI@Li^+^  NPs 50% in SCN | Li | RT | 165 | 50%, 100 cycles,  39.7 mA g^−1^ | 0.5 | Ref. 37 |
|  | PEO gamma-LiAlO_2_,  LiTFSI | Li | 65 ℃ | 183 | 50%, 50 cycles,  0.1 C | 1 | Ref. 38 |
|  | PEO ta-POSS crosslinked,  LiTFSI | Li | 90 ℃ | 245 | 87%, 50 cycles,  0.1 C | 0.18 | Ref. 39 |
|  | PEO, LiTFSI | Li | 100 ℃ | 210 | 75%, 40 cycles,  1.0 C | 0.625 | Ref. 40 |
|  | PEO (LMP) | Li | 100 ℃ | 98 | 65%, 20 cycles,  1.0 C | 1.75 | Ref. 41 |
|  | PEO-LiClO_4_-LLTO | Li | RT | 104 | 90%, 300 cycles,  60 mA g^−1^ | 0.033 | Ref. 42 |
| **Polymer (Gel)** | Crosslinked mPEG-MA,  ionic liquid | poly(TCAQ) | RT | 24 | 87%, 1000 cycles,  1.0 C | 0.023 | Ref. 43 |
| **Inorganic SE** | LPSCl | Li | 60 ℃ | 399 | 75%, 100 cycles,  0.1 C | 0.25 | Ref. 44 |
|  | Na_3_PS_4_ | Na_15_Sn_4_ | 60 ℃ | 275 | 89%, 500 cycles,  0.3 C | 0.022 | Ref. 45 |
|  | 70Li_2_S-30P_2_S_5_ | Li | 60 ℃ | 312 | 90.6%, 500 cycles  0.1 C | 0.019 | Ref. 46 |
|  | Li_7_P_3_S_11_ | Li | 25 ℃ | 410 | 83%, 100 cycles,  60 mA g^−1^ | 0.17 | Ref. 47 |

**Supporting Reference**

1. S. Chandra, T. Kundu, K. Dey, M. Addicoat, T. Heine, R. Banerjee, Interplaying intrinsic and extrinsic proton conductivities in covalent organic frameworks. Chem. Mater*.* **28**, 1489-1494 (2016).
2. J. Evans, C. A. Vincent, P. G. Bruce. Electrochemical measurement of transference numbers in polymer electrolytes. Polymer **28**, 2324 (1987).
3. B. Delley, From molecules to solids with the DMol^3^ approach. J. Chem. Phys*.* **113**, 7756–7764 (2000).
4. J. P. Perdew, K. Burke, M. Ernzerhof, Generalized gradient approximation made simple. Phys. Rev. Lett. **77**, 3865-3868 (2009).
5. A. Tkatchenko, M. Scheffler, Accurate molecular Van Der Waals interactions from ground-state electron density and free-Atom reference data. Phys. Rev. Lett. **102**, 073005 (2009).
6. H. J. Monkhorst, J. D. Pack, Special points for Brillonin-zone integrations. Phys. Rev. B **13**, 5188-5192 (1976).
7. T. A. Halgren, W. N. Lipscomb, The synchronous-transit method for determining reaction pathways and locating molecular transition states. Chem. Phys. Lett. **49***,* 225-232 (1976).
8. K. Jeong, S. Park, G. Y. Jung, S. H. Kim, Y.-H. Lee, S. K. Kwak, S.-Y. Lee, Solvent-free, single lithium-ion conducting covalent organic frameworks. J. Am. Chem. Soc*.* **141**, 5880-5885 (2019).
9. K.-S. Oh, J.-H. Kim, S.-H. Kim, D. Oh, S.-P. Han, K. Jung, Z. Wang, L. Shi, Y. Su, T. Yim, S. Yuan, S.-Y. Lee, Single-ion conducting soft electrolytes for semi-solid lithium metal batteries enabling cell fabrication and operation under ambient conditions. Adv. Energy Mater. **11**, 2101813 (2019).
10. K.-S. Oh, S. Park, J.-S. Kim, Y. Yao, J.-H. Kim, J. Guo, D.-H. Seo, S.-Y. Lee, Electrostatic covalent organic frameworks as on-demand molecular traps for high-energy Li metal battery electrodes. ACS Energy Lett. **8**, 2463-2474 (2023).
11. K.-S. Oh, J. E. Lee, Y.-H. Lee, Y.-S. Jeong, I. Kristanto, H.-S. Min, S.-M. Kim, Y. J. Hong, S. K. Kwak, S.-Y. Lee, Elucidating ion transport phenomena in sulfide/polymer composite electrolytes for practical solid‑state batteries. Nano-Micro Lett.  **15**, 179 (2023).
12. X. Li, Q. Hou, W. Huang, H.-S. Xu, X. Wang, W. Yu, R. Li, K. Zhang, L. Wang, Z. Chen, K. Xie, K. P. Loh, Solution-processable covalent organic framework electrolytes for all-solid-state Li–organic batteries. ACS Energy Lett*.* **5**, 3498-3506 (2020).
13. G. Zhao, Z. Mei, L. Duan, Q. An, Y. Yang, C. Zhang, X. Tan, H. Guo, COF-based single Li^+^ solid electrolyte accelerates the ion diffusion and restrains dendrite growth in quasi-solid-state organic batteries. Carbon Energy **5**, e248 (2023).
14. Y. Sun, G. Zhao, Y. Fu, Y. Yang, C. Zhang, Q. An, H. Guo, Understanding a single-Li-ion COF conductor for being dendrite free in a Li-organic battery. Research DOI: 10.34133/2022/9798582 (2022).
15. R. Bouchet, S. Maria, R. Meziane, A. Aboulaich, L. Lienafa, J. Bonnet, T. N. T. Phan, D. Bertin, D. Gigmes, D. Devaux, R. Denoyel, M. Armand, Single-ion BAB triblock copolymers as highly efficient electrolytes for lithium-metal batteries. Nat. Mater*.* **12**, 452-457 (2013).
16. Y. Zhang, W. Cai, R. Rohan, M. Pan, Y. Liu, X. Liu, C. Li, Y. Sun, H. Cheng, Toward ambient temperature operation with all-solid-state lithium metal batteries with a sp^3^ boron-based solid single ion conducting polymer electrolyte. J. Power Sources **306**, 152-161 (2016).
17. L. Porcarelli, A. S. Shaplov, M. Salsamendi, J. R. Nair, Y. S. Vygodskii, D. Mecerreyes, C. Gerbaldi, Single-ion block copoly(ionic liquid)s as electrolytes for all-solid state lithium batteries. ACS Appl. Mater. Interfaces **8**, 10350-10359 (2016).
18. L. Porcarelli, M. A. Aboudzadeh, L. Rubatat, J. R. Nair, A. S. Shaplov, C. Gerbaldi, D. M. Mecerreyes, Single-ion triblock copolymer electrolytes based on poly(ethylene oxide) and methacrylic sulfonamide blocks for lithium metal batteries. J. Power Sources **364**, 191-199 (2017).
19. H. Yuan, J. Luan, Z. Yang, J. Zhang, Y. Wu, Z. Lu, H. Liu, Single lithium-ion conducting solid polymer electrolyte with superior electrochemical stability and interfacial compatibility for solid-state lithium metal batteries. ACS Appl. Mater. Interfaces **12**, 7249-7256 (2020).
20. Y. Zhang, J. Duan, D. Ma, P. Li, S. Li, H. Li, J. Zhou, X. Ma, X. Feng, B. Wang, Three-dmensional aionic cclodextrin-bsed covalent organic frameworks. Angew. Chem., Int. Ed. **56**, 16313-16317 (2017).
21. J. Li, F.-Q. Zhang, F. Li, Z. Wu, C. Ma, Q. Xu, P. Wang, X.-M. Zhang, A pre-synthetic strategy to construct single ion conductive covalent organic frameworks. Chem. Commun. **56**, 2747-2750 (2020).
22. Y. Hu, N. Dunlap, S. Wan, S. Lu, S. Huang, I. Sellinger, M. Ortiz, Y. Jin, S. Lee, W. Zhang, Crystalline lithium imidazolate covalent organic frameworks with high Li-ion conductivity. J. Am. Chem. Soc. **141**, 7518-7525 (2019).
23. D. A. Vazquez-Molina, G. S. Mohammad-Pour, C. Lee, M. W. Logan, X. Duan, J. K. Harper, J. D. Uribe-Romo, Mechanically shaped two-dimensional covalent organic frameworks reveal crystallographic alignment and fast Li-ion conductivity*.* J. Am. Chem. Soc*.* **138**, 9767-9770 (2016).
24. Q. Xu, S. Tao, Q. Jiang, D. Jiang, Ion conduction in polyelectrolyte covalent organic frameworks. J. Am. Chem. Soc. **140**, 7429-7432 (2016).
25. H. Chen, H. Tu, C. Hu, Y. Liu, D. Dong, Y. Sun, Y. Dai, S. Wang, H. Qian, Z. Lin, Cationic covalent organic framework nanosheets for fast Li-ion conduction. J. Am. Chem. Soc. **140**, 896-899 (2018).
26. G. Zhang, Y.-l. Hong, Y. Nishiyama, S. Bai, S. Kitagawa, S. Horike, Accumulation of glassy poly (ethylene oxide) anchored in a covalent organic framework as a solid-state Li^+^ electrolyte. J. Am. Chem. Soc*.* **141**, 1227-1234 (2018).
27. Z. Li, Z.-W. Liu, Z.-J. Mu, C. Cao, Z. Li, T. X. Wang, Y. Li, X. Ding, B.-H. Han, W. Feng, Cationic covalent organic framework based all-solid-state electrolytes. Mater. Chem. Front. **4**, 1164-1173 (2020).
28. Q. Xu, S. Tao, Q. Jiang, D. Jiang, Designing covalent organic frameworks with a tailored ionic interface for ion transport across one-dimensional channels. Angew. Chem. Int. Ed*.* **59**, 4557-4563 (2020).
29. Z. Li, Z. Liu, Z. Li, T. X. Wang, F. Zhao, X. Ding, W. Feng, B.-H. Han, Defective 2D covalent organic frameworks for postfunctionalization. Adv. Funct. Mater. **30**, 1909267 (2020).
30. Z. Shan, M. Wu, Y. Du, B. Xu, B. He, X. Wu, G. Zhang, Covalent organic framework-based electrolytes for fast Li^+^ conduction and high-temperature solid-state lithium-ion batteries. ACS Appl. Energy Mater. **4**, 11720-11725 (2021).
31. Z. Wang, Y. Zhang, P. Zhang, D. Yan, J. Liu, Y. Chen, Q. Liu, P. Cheng, M. J. Zaworotko, Z. Zhang, Thermally rearranged covalent organic framework with flame-retardancy as a high safety Li-ion solid electrolyte. eScience **2**, 311-318 (2022).
32. W. Gong, Y. Ouyang, S. Guo, Y. Xiao, Q. Zeng, D. Li, Y. Xie, Q. Zhang, S. Huang, Planar chiral multiple resonance thermally activated delayed fluorescence materials for efficient circularly polarized electroluminescence. Angew.Chem. Int. Ed. **62**, e2023025 (2023).
33. Z. Guo, Y. Zhang, Y. Dong, J. Li, S. Li, P. Shao, X. Feng, B. Wang, Fast ion transport pathway provided by polyethylene glycol confined in covalent organic frameworks. J. Am. Chem. Soc. **141**, 1923-1927 (2019).
34. Z. Shan, M. Wu, Y. Du, B. Xu, B. He, X. Wu, G. Zhang, Covalent organic framework-based electrolytes for fast Li^+^ conduction and high-temperature solid-state lithium-ion batteries. Chem. Mater. **33**, 5058-5066 (2021).
35. Z. Zhu, M. Hong, D. Guo, J. Shi, Z. Tao, J. Chen, All-solid-state lithium organic battery with composite polymer electrolyte and pillar[5]quinone cathode. J. Am. Chem. Soc. **136**, 16461-16464 (2014).
36. H. Fei, Y. Liu, Y. An, X. Xu, G. Zheng, Y. Tian, L. Ci, B. Xi, S. Xiong, J. Feng, Stable all-solid-state potassium battery operating at room temperature with a composite polymer electrolyte and a sustainable organic cathode. J. Power Sources **399**, 294-298 (2018).
37. B. Kim, H. Kang, K. Kim, R.-Y. Wang, M. J. Park, All-solid-state lithium–organic batteries comprising single-ion polymer nanoparticle electrolytes. ChemSusChem **13**, 2271-2279 (2020).
38. W. Li, L. Chen, Y. Sun, C. Wang, Y. Wang, Y. Xia, All-solid-state secondary lithium battery using solid polymer electrolyte and anthraquinone cathode. Solid State Ion. **300**, 114-119 (2017).
39. Y. Shi, Y. Chen, Y. Liang, J. Andrews, H. Dong, M. Yuan, W. Ding, S. Banerjee, H. Ardebili, M. L. Robertson, X. Li, Y. Yao, Chemically inert covalently networked triazole-based solid polymer electrolytes for stable all-solid-state lithium batteries. J. Mater. Chem. A **7**, 19691-19695 (2019).
40. M. Lécuyer, J. Gaubicher, A.-L. Barrès, F. Dolhem, M. Deschamps, D. Guyomard, P. Poizot, A rechargeable lithium/quinone battery using a commercial polymer electrolyte. Electrochem. Commun. **55**, 22-25 (2015).
41. M. Lécuyer, M. Deschamps, D. Guyomard, J. Gaubicher, P. Poizot, Assessment of indigo carmine dye in lithium metal polymer technology. Molecules **26**, 3079 (2021).
42. W. Wei, L. Li, L. Zhang, J. Hong, G. He, An all-solid-state Li-organic battery with quinone-based polymer cathode and composite polymer electrolyte. Electrochem. Commun. **90**, 21-25 (2015).
43. S. Muench, R. Burges, A. Lex-Balducci, J. C. Brendel, M. Jäger, C. Friebe, A. Wild, U. S. Schubert, Printable ionic liquid-based gel polymer electrolytes for solid state all-organic batteries. Energy Storage Mater*.* **25**, 750-755 (2020).
44. J. Zhang, Z. Chen, Q. Ai, T. Terlier, F. Hao, Y. Liang, H. Guo, J. Lou, Y. Yao, Microstructure engineering of solid-state composite cathode via solvent-assisted processing*.* Joule **5**, 1845-1859 (2020).
45. F. Hao, X. Chi, Y. Liang, Y. Zhang, R. Xu, H. Guo, T. Terlier, H. Dong, K. Zhao, J. Lou, Y. Yao, Taming active material-solid electrolyte interfaces with organic cathode for all-solid-state batteries. Joule **3**, 1349-1359 (2019).
46. X. Zhou, Y. Zhang, M. Shen, Z. Fang, T. Kong, W. Feng, Y. Xie, F. Wang, B. Hu, Y. Wang, A highly stable Li-organic all-solid-state battery based on sulfide electrolytes. Adv. Energy Mater. **12**, 2103932 (2022).
47. Z. Yang, F. Wang, Z. Hu, J. Chu, H. Zhan, X. Ai, Z. Song. Room-temperature all-solid-state Lithium–organic batteries based on sulfide electrolytes and organodisulfide cathodes. Adv. Energy Mater. **11**, 2102962 (2021).
